# Dual‐input tracer kinetic modeling of dynamic contrast‐enhanced MRI in thoracic malignancies

**DOI:** 10.1002/acm2.12740

**Published:** 2019-10-11

**Authors:** Sang Ho Lee, Andreas Rimner, Joseph O. Deasy, Margie A. Hunt, Neelam Tyagi

**Affiliations:** ^1^ Department of Medical Physics Memorial Sloan Kettering Cancer Center New York NY USA; ^2^ Department of Radiation Oncology Memorial Sloan Kettering Cancer Center New York NY USA

**Keywords:** DCE‐MRI, dual blood supply, malignant pleural mesothelioma, nonsmall cell lung cancer, tracer kinetic modeling

## Abstract

Pulmonary perfusion with dynamic contrast‐enhanced (DCE‐) MRI is typically assessed using a single‐input tracer kinetic model. Preliminary studies based on perfusion CT are indicating that dual‐input perfusion modeling of lung tumors may be clinically valuable as lung tumors have a dual blood supply from the pulmonary and aortic system. This study aimed to investigate the feasibility of fitting dual‐input tracer kinetic models to DCE‐MRI datasets of thoracic malignancies, including malignant pleural mesothelioma (MPM) and nonsmall cell lung cancer (NSCLC), by comparing them to single‐input (pulmonary or systemic arterial input) tracer kinetic models for the voxel‐level analysis within the tumor with respect to goodness‐of‐fit statistics. Fifteen patients (five MPM, ten NSCLC) underwent DCE‐MRI prior to radiotherapy. DCE‐MRI data were analyzed using five different single‐ or dual‐input tracer kinetic models: Tofts‐Kety (TK), extended TK (ETK), two compartment exchange (2CX), adiabatic approximation to the tissue homogeneity (AATH) and distributed parameter (DP) models. The pulmonary blood flow (BF), blood volume (BV), mean transit time (MTT), permeability‐surface area product (PS), fractional interstitial volume (*v*_I_), and volume transfer constant (*K* ^Trans^) were calculated for both single‐ and dual‐input models. The pulmonary arterial flow fraction (*γ*), pulmonary arterial blood flow (BF_PA_) and systemic arterial blood flow (BF_A_) were additionally calculated for only dual‐input models. The competing models were ranked and their Akaike weights were calculated for each voxel according to corrected Akaike information criterion (cAIC). The optimal model was chosen based on the lowest cAIC value. In both types of tumors, all five dual‐input models yielded lower cAIC values than their corresponding single‐input models. The 2CX model was the best‐fitted model and most optimal in describing tracer kinetic behavior to assess microvascular properties in both MPM and NSCLC. The dual‐input 2CX‐model‐derived BF*_A_* was the most significant parameter in differentiating adenocarcinoma from squamous cell carcinoma histology for NSCLC patients.

## 1 INTRODUCTION

Dynamic contrast‐enhanced magnetic resonance imaging (DCE‐MRI) is a well‐established imaging technique for the estimation of tissue microvascular function in clinical settings.^1^, ^2^, ^3^, ^4^, ^5^ To obtain DCE‐MRI data, a contrast agent (CA) is injected into the patient and multiple MR images are acquired at the same spatial location over a time period of approximately five minutes. The temporal passage of CA through tissue reflects its microcirculation and can be used to assess and map out differences in tracer kinetic parameters measuring blood flow, vascular permeability, and tissue volume fractions. The enhancement curve pattern provides relevant information about the biological behavior of tumors.^6^

Lung cancer is the leading cause of cancer‐related deaths in the majority of countries.^7^ Nonsmall cell lung cancer (NSCLC) accounts for approximately 80% of all lung cancers whereas malignant pleural mesothelioma (MPM) is a rare and highly lethal tumor affecting the lining around the lungs and is usually associated with asbestos exposure.^8^, ^9^ Malignant pleural mesothelioma occurs in any part of the visceral pleura that covers the lungs and the parietal pleura that lines the inner surfaces of the chest wall of the pleural cavity; about 80% occurs in the visceral pleura and 20% occurs in the parietal pleura.^10^ The blood supply of the parietal pleura emanates from systemic sources^11^, ^12^: intercostal arteries, the subclavian and internal thoracic arteries, and phrenic arteries. The visceral pleura derives its arterial blood supply from bronchial arterial circulation and from the pulmonary arteries which arise beneath the pleura from the pulmonary circulation.^11^, ^13^ Lung tumors may also have a dual blood supply, due to the pulmonary and aortic systems, with a circulatory pattern that is specific to their histologic types.^14^ Previous studies have performed a single‐input pulmonary or aortic perfusion computed tomography (PCT),^15^, ^16^, ^17^, ^18^, ^19^ DCE‐MRI assessment^20^, ^21^ or, in contrast to the above studies, which measured the arterial input function (AIF), a few DCE‐MRI studies used a reduced model, e.g., Brix model in MPM, where the model assumed a predefined AIF.^22^, ^23^ Recent studies using PCT have reported that a dual‐input maximum (or steepest) slope analysis is valuable in NSCLC.^24^, ^25^, ^26^, ^27^ The dual‐input implementation identified the proportion of the pulmonary (or systemic) arterial perfusion to the total perfusion in the lung tissue, and indicated that perfusion index derived from dual‐input maximum slope PCT analysis has potential to be an important biomarker for thoracic malignancies.

To date, there have been no DCE‐MRI lung studies using dual‐input tracer kinetic models. The dual‐input method of DCE‐MRI has already been applied to liver perfusion studies to separately evaluate the hepatic arterial and portal venous perfusion.^28^, ^29^ However, application of the dual‐input method to lung tumors is challenging because the time difference between the pulmonary and systemic arterial circulations is small (within several seconds). This may result in similar temporal enhancement pattern of the CA between the two arteries as compared with that between the hepatic artery and the portal vein in the liver where the time difference is of the order of 10–20 s. In this study, we sought out to extend the previously developed dual‐input tracer kinetic model to lung DCE‐MRI studies.

The aim of this study was to illustrate the feasibility of dual‐input tracer kinetic modeling by comparing single‐ and dual‐input approaches, for the analysis of DCE‐MRI data from two different types of thoracic malignancies (MPM and NSCLC), and to compare five different tracer kinetic models for the voxel‐level analysis of DCE‐MRI data with respect to goodness‐of‐fit statistics in MPM and NSCLC.

## 2 METHODS AND MATERIALS

### 2.1 Dual‐input tracer kinetic modeling of lung tumor perfusion

A detailed analysis of the model has been described in the supplementary document (supplementary document 1) and in part in our previous publications.^30^ This work considered dual‐input sources for the plasma flow *F* (in mL/min) to the lung tissue, i.e., flow from the pulmonary artery *F*_PA_ and flow from the aorta (representing bronchial artery, intercostal artery, etc.) *F*_A_. Denoting the CA blood concentration‐time curves in the pulmonary artery and aorta as *C*_PA_(*t*) and *C*_A_(*t*) (in g/mL), respectively, CA concentration in the lung tissue *C*_T_(*t*), can be expressed as.

(1) $$\begin{array}{cc} C_{T}\left(t\right) & =R_{T}\left(t-t_{\text{Lag},T}\right)⊗\frac{\left(F_{\text{PA}}/V_{T}\right)C_{\text{PA}}\left(t\right)+\left(F_{A}/V_{T}\right)C_{A}\left(t\right)}{1-H_{\text{LV}}}\\ =\frac{F}{V_{T}}R_{T}\left(t-t_{\text{Lag},T}\right)⊗\frac{\gamma C_{\text{PA}}\left(t\right)+\left(1-\gamma \right)C_{A}\left(t\right)}{1-H_{\text{LV}}}\\ =Q_{T,\text{PA}}\left(t-t_{\text{Lag},T}\right)⊗\frac{C_{\text{PA}}\left(t\right)}{1-H_{\text{LV}}}+Q_{T,\text{PA}}\left(t-t_{\text{Lag},T}\right)⊗\frac{C_{A}\left(t\right)}{1-H_{\text{LV}}}\\ =Q_{T}\left(t-t_{\text{Lag},T}\right)⊗\frac{C_{\text{in}}\left(t\right)}{1-H_{\text{LV}}} \end{array}$$

where *V*_T_ is the volume of tissue (in mL), $F_{\text{PA}}/V_{T}$, $F_{A}/V_{T}$ and $F/V_{T}=\left(F_{\text{PA}}+F_{A}\right)/V_{T}$ are the pulmonary arterial perfusion, systemic arterial perfusion and total pulmonary perfusion (in mL/min/mL), respectively, $\gamma =F_{\text{PA}}/F$ is the pulmonary arterial flow fraction, $H_{\text{LV}}$ is the hematocrit of blood in major vessels (≅ 0.45)^31^, $t_{\text{Lag},T}$ is the difference in bolus arrival time (in min) between *C*_PA_(*t*) (or *C*_A_[*t*]) and *C*_T_(*t*), *R*_T_(*t*) is the tissue residue function (TRF), which is the object for tracer kinetic modeling. The pulmonary impulse response functions (IRF) of the tissue (in mL/min/mL) is $Q_{T,\text{PA}}\left(t\right)=\left(F_{\text{PA}}/V_{T}\right)R_{T}\left(t\right)$, $Q_{T,A}\left(t\right)=\left(F_{A}/V_{T}\right)R_{T}\left(t\right)$ is the systemic IRF of the tissue (in mL/min/mL), $Q_{T}\left(t\right)=\left(F/V_{T}\right)R_{T}\left(t\right)$ is the total IRF of the tissue (in mL/min/mL), and $C_{\text{in}}\left(t\right)$ is the net arterial input function (AIF). The convolution operation ⊗ in Eq. (1) can be visualized as a reflection of *Q*_T_(*t*) about *t* = 0 and by summing the overlapping areas of the reflected *Q*_T_(*t*) and $C_{\text{in}}\left(t\right)$ as the reflected *Q*_T_(*t*) is shifted along the positive *t* direction. Thus, the IRF *Q*_T_(*t*) describes the modeled time‐resolved proportion of retained CA in the tissue as a result of giving one unit‐amplitude of an infinitely narrow bolus in the arterial inlet. Furthermore, different tracer kinetic models have different mathematical forms of IRF due to their different physiologic scenarios in the capillary‐tissue system. Please note that like previous dual‐input kinetic modeling studies for the liver,^28^, ^29^ a single lag time ($t_{\text{Lag},T}$) was used for both pulmonary and systemic arterial delays to the tissue. Because the dual arterial inputs join in the capillary bed, they can effectively be replaced by a single net input function with their mixed contributions (i.e., weighted sum of the dual AIFs), and thereby a single IRF can appear as shown in Eq. (1). Thus, $C_{T}\left(t\right)$ for single‐input models can be derived either from $C_{\text{in}}\left(t\right)=C_{\text{PA}}\left(t\right)$ with γ=1 for the pulmonary AIF or from $C_{\text{in}}\left(t\right)=C_{A}\left(t\right)$ with γ=0 for the systemic AIF.

Because there is always a trade‐off between the complexity of the model and the estimation of model parameters from real‐world data,^32^ five different single‐ or dual‐input tracer kinetic models ranging from simple to complex: the Tofts‐Kety (TK), extended TK (ETK), two compartment exchange (2CX), adiabatic approximation to the tissue homogeneity (AATH), and distributed parameter (DP) models were compared (See supplementary document 1). Notations of various parameters used in this study are provided in supplementary document 2.

### 2.2 Patients

This prospective study was approved by the institutional review board, and all patients have given written informed consent. In brief, 15 patients with single primary thoracic malignancies (five MPM, 10 NSCLC) were included in this study. Ten NSCLC patients were further grouped into adenocarcinoma (AC) and squamous cell carcinoma (SCC) histologies. These patients underwent MRI scans as a part of their radiation therapy simulation scan. The patient group included 3 men and 2 women (age range, 59–77 yr; mean age, 69.6 yr) in MPM group, and 6 men and 4 women (age range, 51–89 yr; mean age, 72 yr) in NSCLC group. All MPM patients had epithelioid histologies. Out of ten NSCLC patients, five were AC histology (stage I or II = 2, stage III or IV = 3) and five were SCC histology (stage I or II = 1, stage III or IV = 4).

### 2.3 Imaging protocol

MR image acquisition was performed on a 3T Philips Ingenia scanner. DCE‐MRI was obtained using a coronal four‐dimensional (4D) T1‐weighted high‐resolution imaging method with volume excitation (4D THRIVE) sequence during free breathing. The 4D THRIVE uses contrast‐enhanced timing robust acquisition, k‐space order technique and parallel imaging to achieve higher spatiotemporal resolution.^33^, ^34^ Fat suppression was turned off to achieve high temporal resolution in the 4D THRIVE sequence. For DCE‐MRI of the five NSCLC patients, a total of 130 dynamic sequences were obtained with 30 coronal slices that had 2.5 mm slice thickness, TR/TE of 4.2/1.9 ms, flip angle (FA) of 15°, in‐plane acquisition resolution of 2.5 mm, and temporal resolution of 2 s. For DCE‐MRI of the five MPM patients, because of the extensive spread of the disease in the entire lung, a temporal resolution of 5 s was attempted for all the patients with TR/TE of 4.2/1.9 ms, FA of 15° and in‐plane acquisition resolution of 2.5 mm. Among these, three patients were acquired with a slice thickness of 3 mm, and the other two with slice thicknesses of 5 mm and 10 mm to cover the entire disease while keeping the same temporal resolution. The bolus injected with a power injector at a rate of 2 mL/s was 0.2 mL/kg of gadolinium‐diethylenetriaminepentaacetic acid (Gd‐DTPA, Magnevist) followed by saline flush. Prior to DCE‐MRI acquisition, 3D coronal fast‐field echo T1‐weighted images with five different FA scans (5, 15, 20, 25 and 30°) with the same orientation and field of view as the dynamic scan were acquired to generate T1 values of these tumors. The native T1 value for each voxel was estimated based on the multiple FA method. T1 at each time point in the dynamic series was determined and the concentration of CA was determined from the change in T1 by assuming the longitudinal relaxivity of the CA to be 4.5 s^−1^mM^−1^ at 3 T.^35^, ^36^, ^37^

### 2.4 Image processing and analysis

A groupwise image registration method implemented in elastix was used for compensating any potential misalignment in DCE‐MRI. The method was based on principal component analysis and made use of the fact that intensity changes in DCE‐MRI can be described using a low‐dimensional signal model.^38^, ^39^ In such an approach, all DCE‐MRI time‐points were simultaneously registered to a mean space. Then, precontrast T1‐weighted images acquired at multiple FAs were also registered to the first set of DCE‐MR images by using 3D translation, rigid, affine and b‐spline deformable registration methods. Effect of motion compensation on DCE‐MRI metrics was also evaluated.

**Figure 1:**
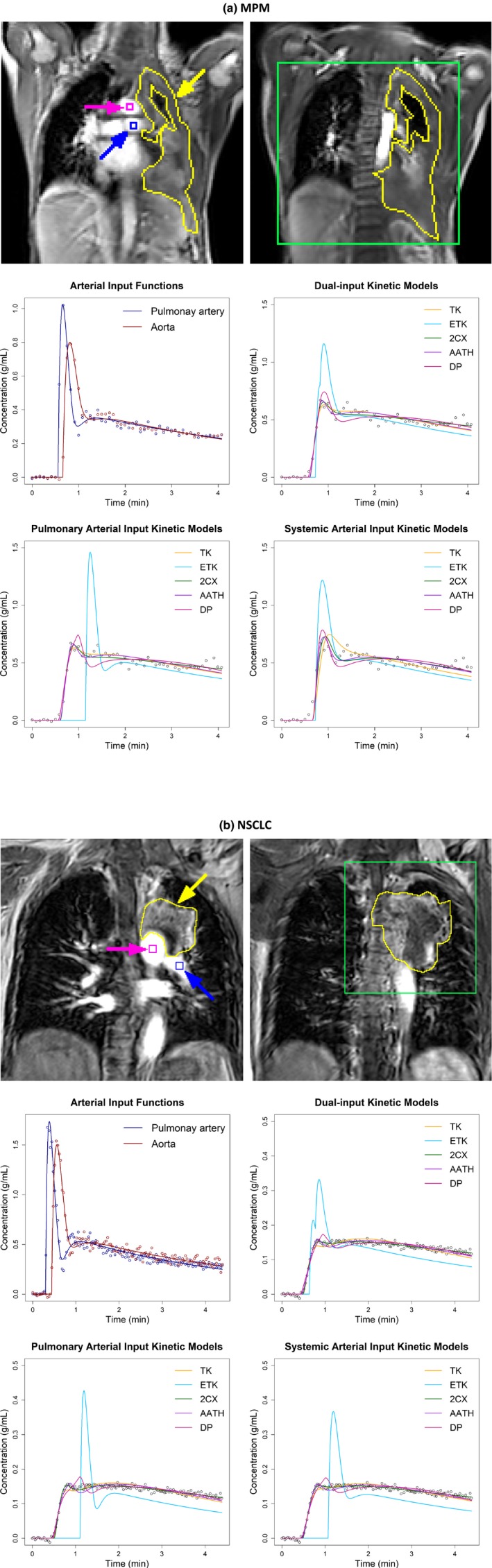
Two central slices of (a) a malignant pleural mesothelioma (MPM) patient and (b) a nonsmall cell lung cancer (NSCLC) patient showing manual outlines of tumors (yellow) and a rectangular window (green) over the tumors used to generate parameter maps with pulmonary, systemic and dual arterial input conditions in Figures 2, 3, 4, respectively. The blue and magenta squares indicate the locations for sampling the pulmonary arterial and aortic input concentration‐time curves, respectively. Graphs illustrate examples of fitting the dual arterial input curves (middle left) and fitting the five different models with dual arterial input function (AIF) (middle right), pulmonary AIF only (lower left) and systemic AIF only (lower right) to voxel‐level tissue concentration‐time curves, which were sampled from (a) the MPM and (b) NSCLC, respectively. TK – Tofts‐Kety, ETK – extended TK, 2CX – two compartment exchange, AATH – adiabatic approximation to the tissue homogeneity, and DP – distributed parameter.

To derive dual‐input curves and delineate tumor margins, regions of interest (ROIs) were placed by an experienced radiation oncologist. To mitigate any potential error in the measurement of AIF, the following criteria for arterial input ROI selection were used – (a) the dual AIF was measured on the main pulmonary and aorta near central slices in the image volume, (b) bolus arrival time was earlier in the pulmonary artery than in the aorta, (c) pulmonary peak concentration was higher than aortic peak concentration, and (d) pulmonary and aortic concentrations in the delayed washout phase were at about the same level. The same dual‐input curves, which were extracted from the ROIs in the pulmonary trunk (or the main left/right pulmonary artery) and in the aorta at the level between the aortic arch and descending aorta for each patient, were used for all kinetic models. For fitting the dual‐input curves, Lee’s AIF model was adopted.^29^ The model led to a new form of analytic solution of *C*_T_(t) by imposing the recirculation delay in addition to the onset time of the first‐pass bolus upon Orton’s AIF model^40^ (see supplementary document 1). This resulted in an AIF model that was the superposition of the first and second pass of the bolus and fitted at the same time to the full‐pass data of the arterial concentration‐time curve. A freehand ROI was carefully drawn to generate concentration‐time curves in tumor volume. All model parameters were calculated using the BOBYQA nonlinear optimization algorithm,^41^ which minimizes the sum squared difference between model fit and data. The following parameters were computed for each single‐ or dual‐input model: pulmonary blood flow (BF, mL/min/100 g), blood volume (BV, mL/100 g), mean transit time (MTT, min), permeability‐surface area product (PS, mL/min/100 g), fractional interstitial volume (*v*_I_), and volume transfer constant ($K^{\text{Trans}}$, mL/min/mL). For each dual‐input model, pulmonary arterial flow fraction (*γ*), pulmonary arterial blood flow (BF_PA_, mL/min/100 g) and systemic arterial blood flow (BF_A_, mL/min/100 g) were also calculated. Examples of the fitting of a voxel‐level concentration‐time curve in both MPM and NSCLC are shown in Figure 1.

### 2.5 Model comparison

The parameterization rules described by Lee, et al. for fair comparisons among different models were employed by using the same number of fitting parameters (i.e., $F/V_{P}$, PS/*V*_P_, $v_{P}$, $v_{I}$ and $t_{\text{Lag},T}$ for single‐input models and along with an additional parameter *γ* for dual‐input models) (see supplementary document 1).^29^ Typically, TK and ETK models have fewer parameters because *K* ^Trans^ is considered a lumped parameter. In our analysis, *K* ^Trans^ was decomposed into *E* and $F/V_{T}$ (and thereby into *F* and PS) under a mixed flow‐ and permeability‐limited condition for the TK and ETK models with a modified assumption that $v_{P}\ll v_{I}$, but $v_{P}\neq 0$ for the TK model so that the five models had the same complexity in the number of fitting parameters.^29^

To compare the fitting quality of the five different single‐ or dual‐input tracer kinetic models, the same approach described by Brix, et al. was used.^42^ For each voxel, Akaike information criterion (AIC) was calculated and corrected for small sample sizes (cAIC).^42^ Based on the cAIC values, the optimal model was chosen by selecting the minimum cAIC (cAIC_min_). The relative strength of support for each of the five models was quantified by Akaike weight (*w_m_*), which expresses the probability for a model to be the best among a set of models. To assess the clinical significance of single‐ or dual‐input kinetic modeling, the intratumoral median values of kinetic parameters were compared between AC and SCC histologies for NSCLC patients for each model.

### 2.6 Statistical analysis

On all analyzed voxels, the following were performed: (a) Pearson’s correlation analysis to detect the linear relationship between the estimates of the same parameter for the five models, (b) Wilcoxon signed rank test to compare differences between the different models, and (c) Wilcoxon rank sum test to determine whether parameters have similar distribution between MPM and NSCLC for each parameter and model. To investigate whether a particular model provides a significantly better fit to the same dataset, cAIC_min_ and *w_m_* of the five models were investigated. All analyses were performed using the statistical software R 3.3.2. A Wilcoxon rank sum test was also performed to test the statistical significance of using single‐ or dual‐input kinetic model parameters in differentiating between AC and SCC histologies. A *P* < 0.05 was considered statistically significant.

## 3 RESULTS

**Figure 2:**
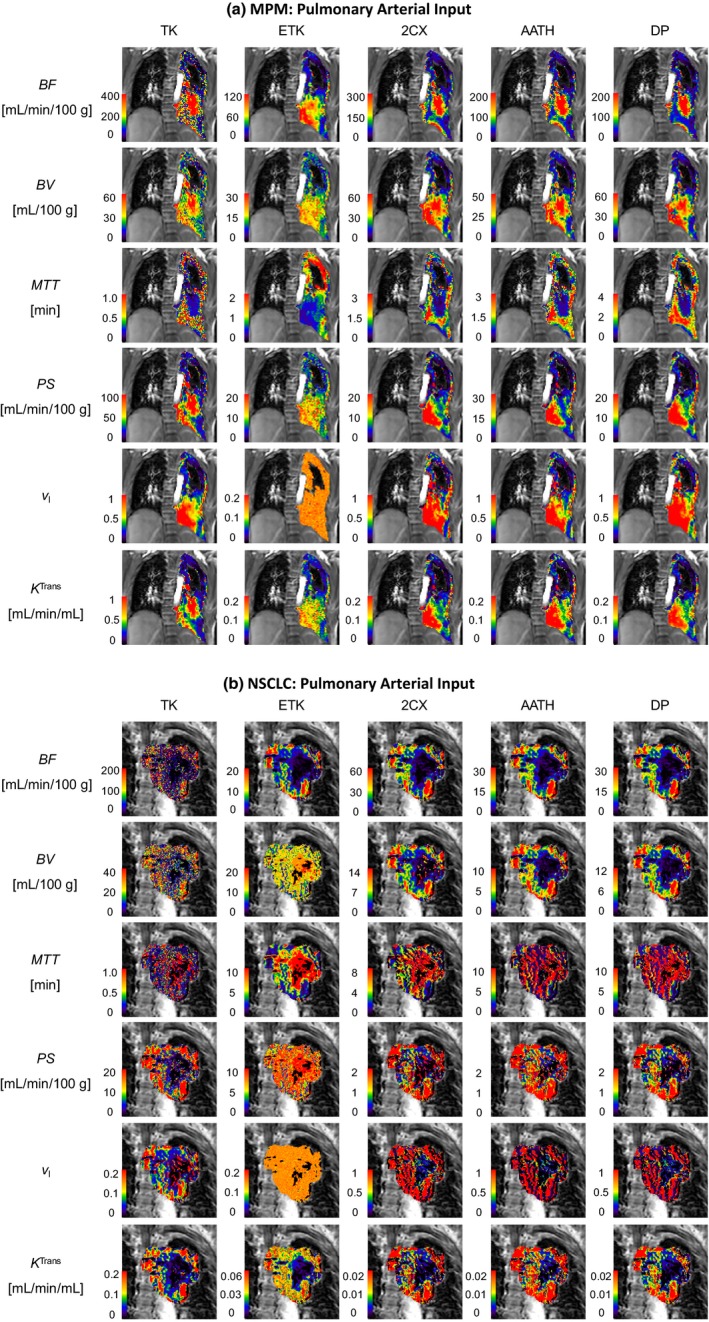
Pulmonary arterial input kinetic parameter maps of the pulmonary blood flow (BF), blood volume (BV), mean transit time (MTT), permeability‐surface area product (PS), fractional interstitial volume ($v_{I}$) and volume transfer constant (*K* ^Trans^) in (a) a malignant pleural mesothelioma patient and (b) a nonsmall cell lung cancer patient. TK – Tofts‐Kety, ETK – extended TK, 2CX – two compartment exchange, AATH – adiabatic approximation to the tissue homogeneity, and DP – distributed parameter.

**Figure 3:**
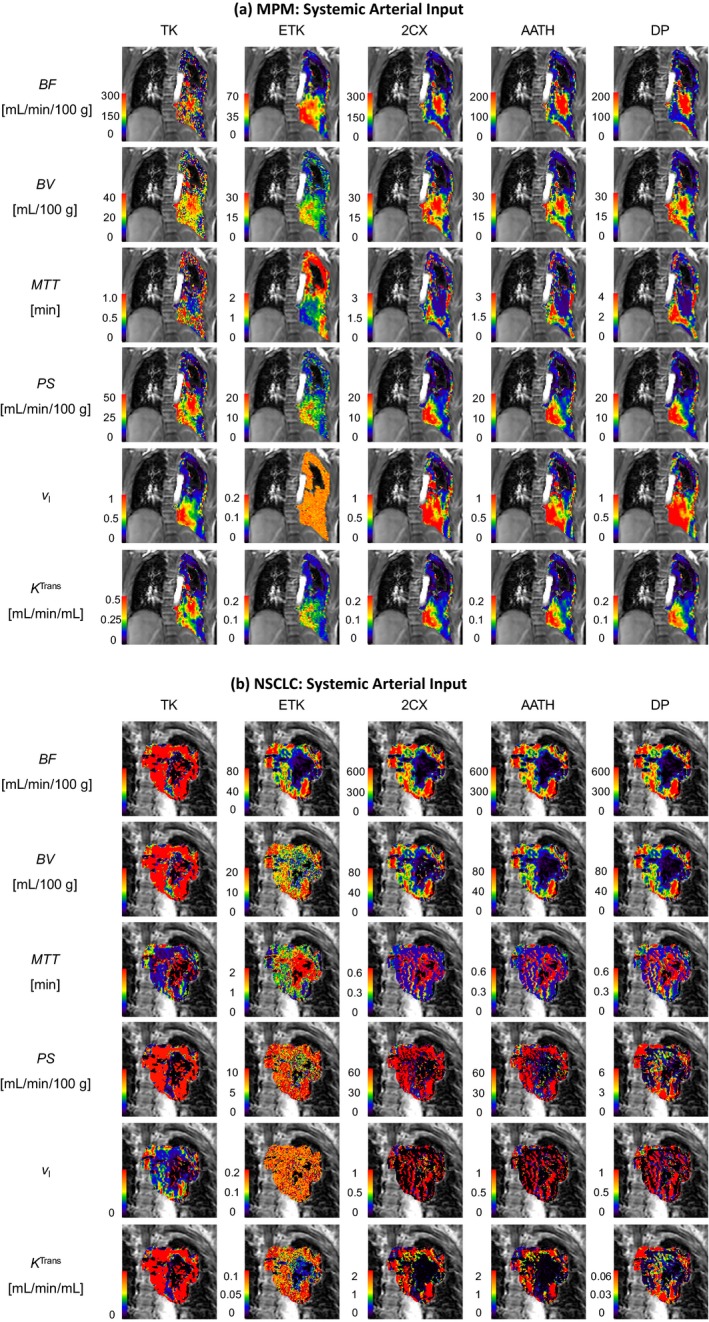
Systemic arterial input kinetic parameter maps of the pulmonary blood flow (BF), blood volume (BV), mean transit time (MTT), permeability‐surface area product (PS), fractional interstitial volume ($v_{I}$) and volume transfer constant (*K* ^Trans^) in (a) a malignant pleural mesothelioma patient and (b) a nonsmall cell lung cancer patient. TK – Tofts‐Kety, ETK – extended TK, 2CX – two compartment exchange, AATH – adiabatic approximation to the tissue homogeneity, and DP – distributed parameter.

**Figure 4:**
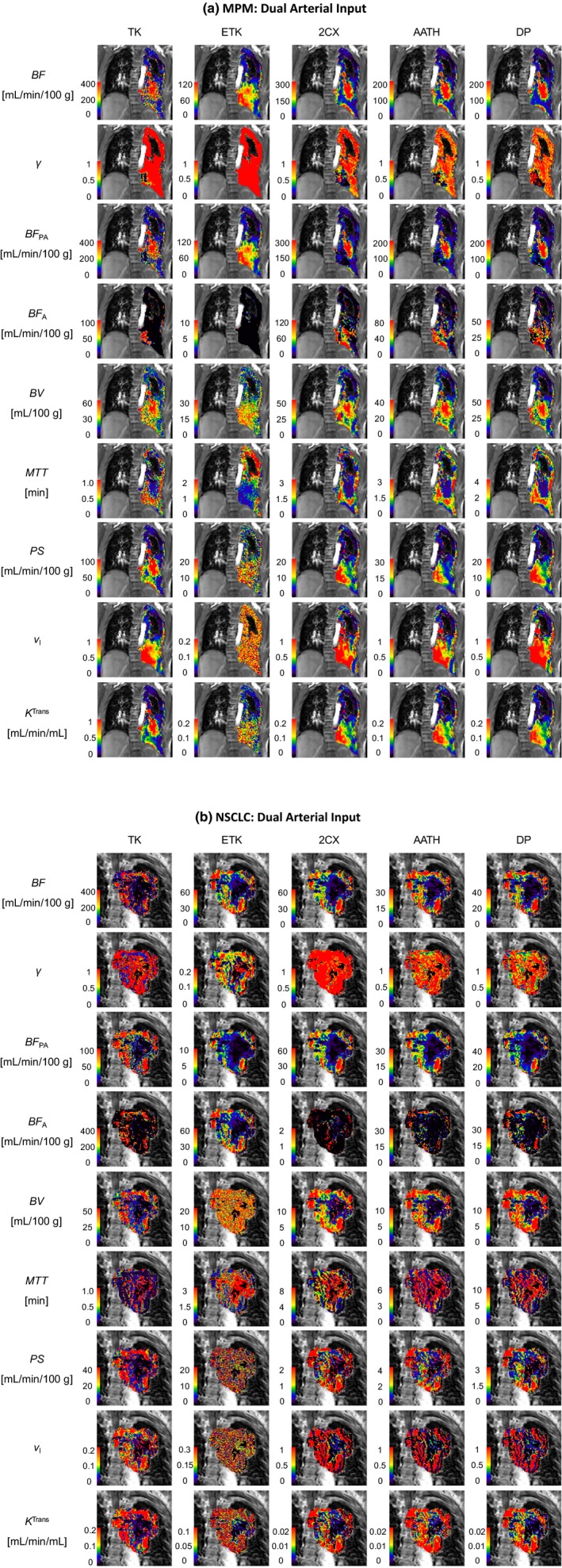
Dual‐input kinetic parameter maps of the total pulmonary blood flow (BF), pulmonary arterial flow fraction (*γ*), pulmonary arterial blood flow (BF_PA_), systemic arterial blood flow (BF_A_), blood volume (BV), mean transit time (MTT), permeability‐surface area product (PS), fractional interstitial volume ($v_{I}$) and volume transfer constant (*K* ^Trans^) in (a) a malignant pleural mesothelioma patient and (b) a nonsmall cell lung cancer patient. TK – Tofts‐Kety, ETK – extended TK, 2CX – two compartment exchange, AATH – adiabatic approximation to the tissue homogeneity, and DP – distributed parameter.

Two examples each for MPM and NSCLC cases are shown in Figs. 2, 3, 4, with voxel‐level fittings and parameter maps generated from the five models with the pulmonary [Figs. 2(a) and 2(b)], systemic [Figs. 3(a) and 3(b)] and dual AIFs [Figure 4(a) and 4(b)], respectively. These parameter maps provide intratumoral spatial information of the various parameters and show their voxel‐level mapping using the single‐ or dual‐input tracer kinetic modeling in lung DCE‐MRI. Significant heterogeneity is observed in all parameter maps in both types of tumors. The spatial heterogeneity, in maps of the same parameter, differs between models, indicating variabilities in different models.

**Figure 5:**
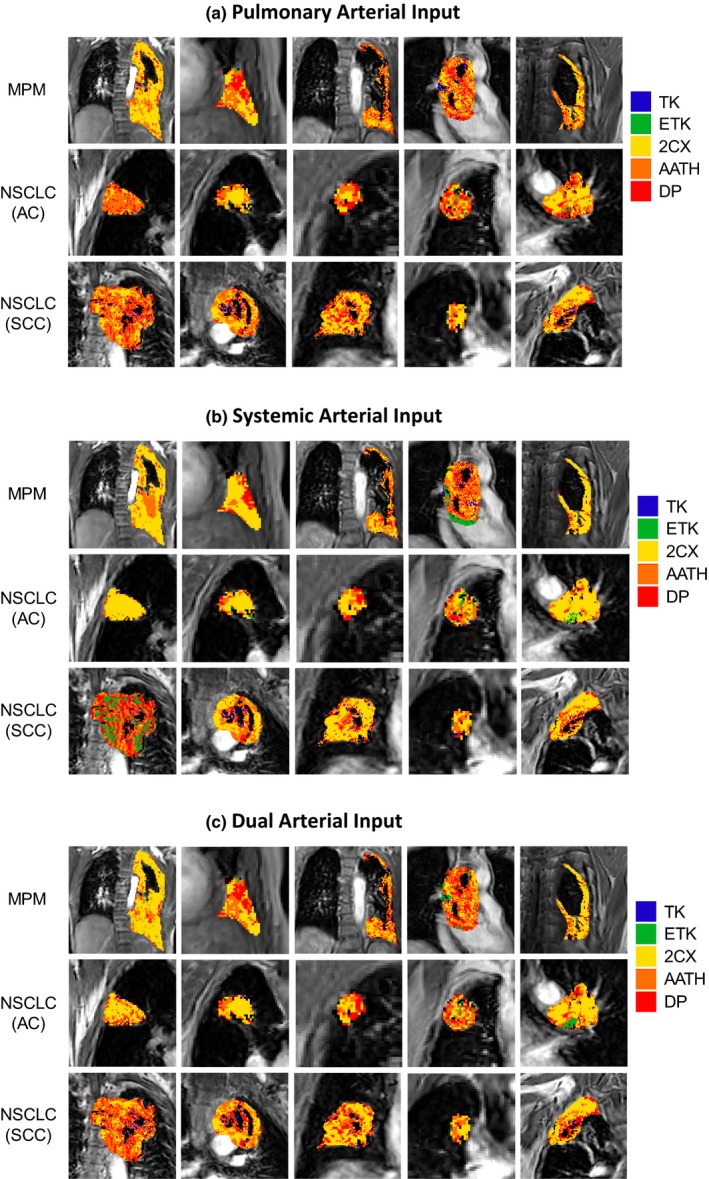
Central tumor slices of minimum corrected Akaike information criterion (cAIC_min_) in five malignant pleural mesothelioma (MPM) patients (upper row) and 10 nonsmall cell lung cancer (NSCLC) patients consisting of five adenocarcinoma (AC) patients (middle row) and five squamous cell carcinoma (SCC) patients (lower row). The cAIC_min_ map reflects in what regions the Tofts‐Kety (TK), extended TK (ETK), two compartment exchange (2CX), adiabatic approximation to the tissue homogeneity (AATH) and distributed parameter (DP) models were the optimal tracer kinetic model in terms of goodness‐of‐fit, with (a) pulmonary, (b) systemic and (c) dual arterial input conditions, respectively.

Figure 5 shows the central tumor slice representing cAIC_min_ for all 5 MPM and 10 NSCLC (i.e., 5 AC and 5 SCC) patients. The map illustrates that the best‐fitting model differs in between contiguous regions within tumor. For individual MPM patients, the TK model was optimal in 1.15–7.49% of voxels with pulmonary AIF, 0.29–8.05% with systemic AIF and 0.01–2.87% with dual AIF. The corresponding % of voxels with pulmonary AIF, systemic AIF and dual AIF for ETK, 2CX, AATH and DP models are (0.08–2.88%, 0.08–4.79%, 0.19–6.35%); (22.1–65.8%, 21.1–71.2%, 23.2–77.5%); (24.0–57.1%, 16.9–42.4%, 15.7–39.8%); (8.90–33.6%, 4.95–26.6%, 5.96–34.8%), respectively. For NSCLC patients, % voxels with pulmonary AIF, systemic AIF and dual AIF for TK, ETK, 2CX, AATH and DP models include (0.76–5.06%, 0.53–13.2%, 0.26–4.19%); (0.07–8.96%, 0.16–31.8%, 0.12–19.0%); (13.9–53.8%, 3.40–80.2%, 26.3–59.0%); (27.8–61.0%, 12.0–35.9%, 22.9–44.3%); (14.2–28.8%, 4.76–37.6%, 10.4–31.9%), respectively.

Percentage for each model to have cAIC_min_ on all the voxels was analyzed in both MPM and NSCLC. For the 58,367 voxels analyzed in MPM, the TK model was optimal in 4.37% of voxels with pulmonary AIF, 3.53% of voxels with systemic AIF and 1.38% of voxels with dual AIF. The optimal % of voxels in ETK, 2CX, AATH and DP models for pulmonary AIF, systemic AIF and dual AIF were (1.26%, 2.16%, 2.86%); (34.3%, 51.4%, 46%); (41.6%, 28.3%, 31.1%); (18.4%, 14.6%, 18.16%), respectively. For all the 165,864 voxels analyzed in NSCLC, the TK model was optimal in 3.20% with pulmonary AIF, 2.05% with systemic AIF and 2.08% with dual AIF. The optimal % of voxels in ETK, 2CX, AATH and DP models for pulmonary AIF, systemic AIF and dual AIF were (0.36%, 10%, 0.91%); (38.9%, 42.5%, 42.4%); (38.8%, 26%, 35.4%); (18.8%, 19.5%, 19.2%), respectively. As a result, the 2CX model was most frequently chosen as the optimal model with the single or dual AIF, followed by AATH, DP, TK and ETK models in both types of tumors.

Tables 1, 2, 3 summarize the parameter values observed in all the voxels in both MPM and NSCLC when calculated with the five models with pulmonary, systemic and dual AIFs, respectively. Overall, BF was closer between pulmonary arterial input and dual‐input kinetic models than between systemic arterial input and dual‐input kinetic models. In comparison between BF_PA_ and BF_A_, BF_PA_ was higher than BF_A_ for the dual‐input 2CX, AATH and DP models in both types of tumors, whereas it was higher for the dual‐input TK model only in NSCLC and higher for the dual‐input ETK model only in MPM. However, BF_A_ was not negligible for all five models. In general, the highest median Akaike weight or *w_m_* was found for the 2CX model followed by the AATH and DP models in both types of tumors, although the median $w_{m}$ for the AATH model was slightly higher than that for the 2CX model in MPM with pulmonary AIF. All voxel‐level parameter values were statistically significantly different (*P* < 0.01) in the pairwise comparison between adjacent different kinetic models (i.e., TK vs. ETK; ETK vs. 2CX; 2CX vs. AATH and AATH vs. DP). The distributions of all model parameter values were statistically significantly different (*P* < 0.01) between MPM and NSCLC with a single or dual AIF.

**Table 1:** Median with 95% confidence interval of tracer kinetic parameters derived from fitting the five pulmonary arterial input tracer kinetic models on all analyzed voxels within MPM (n = 58,367) and NSCLC (n = 165,864).

Parameter	Lesion	TK		ETK		2CX		AATH		DP
BF (mL/min/100g)	MPM	215.7 (213.5, 217.9)	>	41.84 (41.42, 42.27)	<	115.1 (113.9, 116.3)	>	73.93 (73.10, 74.76)	>	64.28 (63.58, 65.00)
	NSCLC	85.80 (85.17, 86.43)	>	10.25 (10.19, 10.32)	<	27.65 (27.46, 27.84)	>	16.60 (16.49, 16.71)	>	15.96 (15.86, 16.07)
BV (mL/100g)	MPM	32.17 (31.97, 32.38)	>	12.60 (12.54, 12.67)	<	29.77 (29.48, 30.06)	>	23.14 (22.91, 23.37)	<	27.18 (26.90, 27.46)
	NSCLC	20.48 (20.39, 20.57)	>	11.06 (11.04, 11.08)	>	8.846 (8.792, 8.901)	>	6.373 (6.333, 6.413)	<	7.129 (7.085, 7.174)
MTT (min)	MPM	0.312 (0.309, 0.314)	<	1.159 (1.148, 1.171)	<	1.269 (1.257, 1.281)	<	1.617 (1.603, 1.631)	<	2.149 (2.133, 2.166)
	NSCLC	0.301 (0.299, 0.303)	<	4.836 (4.789, 4.862)	>	2.665 (2.641, 2.688)	>	1.741 (1.729, 1.754)	<	2.459 (2.444, 2.474)
PS (mL/min/100g)	MPM	57.06 (56.28, 57.87)	>	8.276 (8.232, 8.321)	<	13.09 (12.88, 13.31)	<	14.98 (14.77, 15.19)	>	9.886 (9.770, 10.00)
	NSCLC	17.71 (17.58, 17.85)	>	7.482 (7.466, 7.498)	>	2.404 (2.382, 2.425)	<	4.385 (4.352, 4.418)	>	2.443 (2.428, 2.458)
$v_{I}$	MPM	0.454 (0.449, 0.458)	>	0.150 (0.150, 0.150)	<	0.620 (0.617, 0.623)	>	0.569 (0.567, 0.571)	<	0.635 (0.633, 0.638)
	NSCLC	0.118 (0.117, 0.118)	<	0.150 (0.150, 0.150)	<	0.467 (0.461, 0.473)	>	0.151 (0.149, 0.152)	<	0.227 (0.225, 0.230)
*K* ^Trans^ (mL/min/mL)	MPM	0.466 (0.460, 0.473)	>	0.073 (0.072, 0.073)	<	0.110 (0.109, 0.112)	<	0.123 (0.121, 0.125)	>	0.085 (0.084, 0.086)
	NSCLC	0.126 (0.125, 0.127)	>	0.035 (0.035, 0.035)	>	0.021 (0.021, 0.021)	<	0.034 (0.034, 0.034)	>	0.021 (0.021, 0.021)
$w_{m}$	MPM	0.010 (0.009, 0.011)	>	0.000 (0.000, 0.000)	<	0.320 (0.316, 0.325)	<	0.339 (0.336, 0.342)	>	0.200 (0.198, 0.202)
	NSCLC	0.019 (0.018, 0.019)	>	0.000 (0.000, 0.000)	<	0.354 (0.352, 0.357)	>	0.307 (0.306, 0.309)	>	0.216 (0.215, 0.217)

“>” or “<” indicates significant difference (*P* < 0.01) between adjoining models in the Wilcoxon signed rank test.

**Table 2:** Median with 95% confidence interval of tracer kinetic parameters derived from fitting the five systemic arterial input tracer kinetic models on all analyzed voxels within MPM (n = 58,367) and NSCLC (n = 165,864).

Parameter	Lesion	TK		ETK		2CX		AATH		DP
BF (mL/min/100g)	MPM	193.8 (191.7, 195.9)	>	38.33 (37.92, 38.74)	<	115.3 (114.0, 116.5)	>	83.40 (82.50, 84.31)	>	66.19 (65.55, 66.84)
	NSCLC	257.3 (253.9, 260.7)	>	20.87 (20.73, 21.01)	<	88.62 (87.59, 89.67)	>	70.19 (69.24, 71.15)	>	65.89 (64.95, 66.85)
BV (mL/100g)	MPM	30.17 (29.97, 30.36)	>	12.27 (12.21, 12.34)	<	22.94 (22.68, 23.20)	>	17.76 (17.57, 17.96)	<	22.02 (21.76, 22.29)
	NSCLC	37.01 (36.70, 37.33)	>	11.31 (11.29, 11.34)	<	16.50 (16.39, 16.62)	>	12.74 (12.63, 12.84)	<	14.69 (14.57, 14.81)
MTT (min)	MPM	0.327 (0.324, 0.330)	<	1.280 (1.267, 1.293)	>	1.100 (1.087, 1.113)	<	1.224 (1.213, 1.236)	<	1.805 (1.791, 1.820)
	NSCLC	0.216 (0.214, 0.218)	<	2.768 (2.744, 2.792)	>	1.105 (1.094, 1.116)	>	0.927 (0.922, 0.932)	<	1.398 (1.391, 1.405)
PS (mL/min/100g)	MPM	50.80 (50.07, 51.54)	>	8.100 (8.058, 8.143)	<	13.76 (13.56, 13.96)	<	16.24 (16.04, 16.46)	>	10.91 (10.78, 11.04)
	NSCLC	67.83 (66.41, 69.30)	>	7.629 (7.609, 7.649)	>	7.502 (7.399, 7.607)	<	10.11 (10.01, 10.22)	>	3.837 (3.812, 3.861)
$v_{I}$	MPM	0.421 (0.417, 0.426)	>	0.150 (0.150, 0.150)	<	0.575 (0.573, 0.578)	>	0.519 (0.516, 0.522)	<	0.591 (0.589, 0.594)
	NSCLC	0.207 (0.206, 0.208)	>	0.150 (0.150, 0.150)	<	0.319 (0.314, 0.324)	>	0.144 (0.143, 0.146)	<	0.243 (0.241, 0.245)
*K* ^Trans^ (mL/min/mL)	MPM	0.414 (0.408, 0.419)	>	0.070 (0.069, 0.070)	<	0.121 (0.120, 0.123)	<	0.138 (0.137, 0.140)	>	0.095 (0.094, 0.096)
	NSCLC	0.542 (0.531, 0.554)	>	0.047 (0.047, 0.047)	<	0.063 (0.063, 0.064)	<	0.080 (0.080, 0.081)	>	0.034 (0.034, 0.034)
$w_{m}$	MPM	0.010 (0.009, 0.011)	>	0.000 (0.000, 0.000)	<	0.493 (0.490, 0.496)	>	0.251 (0.249, 0.252)	>	0.156 (0.155, 0.158)
	NSCLC	0.005 (0.005, 0.005)	>	0.000 (0.000, 0.000)	<	0.410 (0.407, 0.412)	>	0.218 (0.217, 0.219)	>	0.179 (0.178, 0.180)

“>” or “<” indicates significant difference (*P* < 0.01) between adjoining models in the Wilcoxon signed rank test.

**Table 3:** Median with 95% confidence interval of tracer kinetic parameters derived from fitting the five dual‐input tracer kinetic models on all analyzed voxels within MPM (n = 58,367) and NSCLC (n = 165,864).

Parameter	Lesion	TK		ETK		2CX		AATH		DP
BF (mL/min/100g)	MPM	217.3 (214.9, 219.7)	>	42.13 (41.72, 42.54)	<	117.0 (115.7, 118.3)	>	79.10 (78.25, 79.95)	>	66.15 (65.46, 66.84)
	NSCLC	91.06 (90.25, 91.88)	>	17.59 (17.48, 17.70)	<	29.36 (29.15, 29.58)	>	19.27 (19.13, 19.41)	<	19.36 (19.22, 19.50)
*γ*	MPM	0.501 (0.501, 0.501)	<	0.668 (0.666, 0.669)	>	0.628 (0.624, 0.632)	<	0.748 (0.745, 0.751)	<	0.780 (0.777, 0.783)
	NSCLC	0.779 (0.775, 0.783)	>	0.476 (0.475, 0.478)	<	0.785 (0.783, 0.786)	<	0.832 (0.830, 0.833)	>	0.810 (0.808, 0.812)
BF_PA_ (mL/min/100g)	MPM	71.06 (69.77, 72.36)	>	25.03 (24.76, 25.30)	<	45.78 (45.22, 46.34)	>	39.61 (39.14, 40.08)	>	37.45 (36.98, 37.92)
	NSCLC	53.13 (52.74, 53.52)	>	6.095 (6.043, 6.147)	<	20.45 (20.30, 20.60)	>	13.72 (13.63, 13.81)	>	13.16 (13.07, 13.25)
BF_A_ (mL/min/100g)	MPM	73.54 (71.54, 75.34)	>	11.15 (10.89, 11.40)	<	38.35 (37.48, 39.20)	>	20.76 (20.28, 21.26)	>	16.29 (15.88, 16.68)
	NSCLC	10.40 (10.04, 10.76)	>	7.731 (7.656, 7.808)	>	3.569 (3.510, 3.630)	>	2.314 (2.269, 2.360)	<	2.869 (2.818, 2.922)
BV (mL/100g)	MPM	34.33 (34.08, 34.58)	>	13.19 (13.12, 13.26)	<	26.37 (26.11, 26.63)	>	21.18 (20.97, 21.40)	<	24.11 (23.87, 24.35)
	NSCLC	21.13 (21.03, 21.24)	>	12.64 (12.60, 12.67)	>	8.910 (8.855, 8.964)	>	7.198 (7.154, 7.243)	<	8.307 (8.255, 8.359)
MTT (min)	MPM	0.336 (0.333, 0.339)	<	1.168 (1.155, 1.182)	>	1.159 (1.148, 1.171)	>	1.410 (1.398, 1.422)	<	1.979 (1.964, 1.993)
	NSCLC	0.268 (0.266, 0.269)	<	3.126 (3.101, 3.152)	>	2.315 (2.294, 2.336)	>	1.607 (1.597, 1.617)	<	2.263 (2.252, 2.274)
PS (mL/min/100g)	MPM	58.19 (57.35, 59.05)	>	8.439 (8.373, 8.506)	<	13.81 (13.60, 14.02)	<	15.23 (15.03, 15.44)	>	10.56 (10.44, 10.69)
	NSCLC	21.74 (21.51, 21.97)	>	8.191 (8.153, 8.228)	>	2.795 (2.771, 2.819)	<	4.607 (4.574, 4.640)	>	2.707 (2.691, 2.723)
$v_{I}$	MPM	0.457 (0.453, 0.462)	>	0.153 (0.152, 0.153)	<	0.597 (0.595, 0.600)	>	0.547 (0.545, 0.550)	<	0.620 (0.618, 0.623)
	NSCLC	0.143 (0.143, 0.144)	<	0.154 (0.154, 0.154)	<	0.423 (0.417, 0.428)	>	0.157 (0.155, 0.159)	<	0.234 (0.232, 0.236)
*K* ^Trans^ (mL/min/mL)	MPM	0.478 (0.471, 0.484)	>	0.069 (0.068, 0.069)	<	0.116 (0.114, 0.117)	<	0.127 (0.126, 0.129)	>	0.091 (0.090, 0.092)
	NSCLC	0.184 (0.182, 0.185)	>	0.043 (0.043, 0.044)	>	0.024 (0.024, 0.025)	<	0.036 (0.036, 0.036)	>	0.023 (0.023, 0.023)
$w_{m}$	MPM	0.011 (0.010, 0.011)	>	0.000 (0.000, 0.000)	<	0.450 (0.446, 0.454)	>	0.266 (0.264, 0.268)	>	0.190 (0.189, 0.192)
	NSCLC	0.019 (0.019, 0.020)	>	0.000 (0.000, 0.000)	<	0.385 (0.383, 0.388)	>	0.291 (0.290, 0.292)	>	0.210 (0.209, 0.211)

“>” or “<” indicates significant difference (*P* < 0.01) between adjoining models in the Wilcoxon signed rank test.

Table 4 compares median cAIC values along with their 95% confidence interval derived from fitting each single‐ or dual‐input tracer kinetic model on all analyzed voxels within MPM or NSCLC. Among the five different models, the 2CX model had the lowest cAIC value in both MPM and NSCLC with dual AIF, in NSCLC with pulmonary AIF, and in MPM with systemic AIF. The AATH model had the lowest cAIC value in MPM with pulmonary AIF, and the DP model in NSCLC with systemic AIF. Among the three different arterial input conditions, the dual‐input kinetic modeling approach yielded the lowest cAIC values in all five models in both types of tumors. The ETK model showed positive cAIC values because of poor fits as the model does not account for the effect of CA exchange and dispersion in the intravascular plasma space.

**Table 4:** Median with 95% confidence interval of cAIC values derived from fitting each single‐ (pulmonary or systemic artery) or dual‐input tracer kinetic model on all analyzed voxels within MPM (n = 58,367) or NSCLC (n = 165,864).

Arterial Input	Lesion	TK	ETK	2CX	AATH	DP
Dual	MPM	−18.58 (−19.22, −17.95)	61.37 (60.92, 61.82)	−50.04 (−50.84, −49.25)	−48.92 (−49.69, −48.15)	−44.71 (−45.48, −43.95)
	NSCLC	‐27.72 (−28.64, −26.80)	135.4 (134.5, 136.3)	−55.42 (−56.38, −54.45)	−54.18 (−55.14, −53.23)	−51.24 (−52.19, −50.29)
Single (Pulmonary Artery)	MPM	−15.94 (−16.56, −15.32)	82.47 (81.98, 82.95)	−43.81 (−44.57, −43.05)	−46.81 (−47.57, −46.06)	−41.13 (−41.90, −40.36)
	NSCLC	−23.31 (−24.22, −22.39)	230.5 (229.7, 231.4)	−54.65 (−55.61, −53.69)	−54.16 (−55.11, −53.20)	−50.99 (−51.93, −50.04)
Single (Systemic Artery)	MPM	−7.250 (−7.883, −6.615)	85.60 (85.11, 86.09)	−40.51 (−41.27, −39.74)	−39.02 (−39.76, −38.28)	−33.90 (−34.64, −33.15)
	NSCLC	−7.315 (−8.253, −6.376)	181.1 (180.1, 182.2)	−31.12 (−32.10, −30.14)	−32.21 (−33.17, −31.24)	−32.51 (−33.48, −31.55)

Tables 5, 6, 7 compare the intratumoral median values of kinetic parameters calculated with the five models with pulmonary, systemic and dual AIFs between two NSCLC histologies (i.e., AC and SCC), respectively. The values with *P* < 0.05 and *P* < 0.1 in comparison between AC and SCC are shown in bold. With all five models, BF and *K* ^Trans^ for AC were higher than those for SCC in the pulmonary arterial input condition, $v_{I}$ for AC was higher than that for SCC in the systemic arterial input condition, and BF, BF_PA_ and *K* ^Trans^ for AC were higher than those for SCC in the dual‐input condition. The mean transit time for AC was lower than that for SCC in all three arterial input conditions. With all four models except for the ETK model, BF_A_ for AC was higher than that for SCC. In the pulmonary arterial input condition, no parameters differed statistically significantly between AC and SCC. In the systemic arterial input condition, the DP model‐derived PS (*P* = 0.032), $v_{I}$ (*P* = 0.032) and *K* ^Trans^ (*P* = 0.032) differed statistically significantly between AC and SCC. In the dual‐input condition, only the 2CX‐model‐derived BF_A_ (*P* = 0.016) differed statistically significantly, between AC and SCC. The dual‐input 2CX‐model‐derived BF_A_ was the most significant parameter in differentiating between the two histologies. Marginally significant difference (*P* < 0.1) was found with all five models with pulmonary or dual AIF, and statistically significant difference (*P* < 0.05) with only the dual‐input 2CX and systemic arterial input DP models.

**Table 5:** Population mean of intratumoral median parameter values comparing AC (n = 5) and SCC (n = 5) NSCLC histologies with the five pulmonary arterial input tracer kinetic models.

Parameter	Histology	TK	ETK	2CX	AATH	DP
BF (mL/min/100g)	AC	232.1	23.95†	95.38†	54.89	51.70
	SCC	116.3	11.89†	47.05†	28.61	27.95
BV (mL/100g)	AC	35.97	10.71	21.19†	17.02†	18.80†
	SCC	22.84	11.48	10.17†	7.693†	8.857†
MTT (min)	AC	0.152	1.292†	1.088	1.009	1.532
	SCC	0.236	3.524†	1.877	1.448	2.083
PS (mL/min/100g)	AC	59.81	7.032	3.908	7.071	3.703
	SCC	30.70	7.737	1.791	3.494	2.057
$v_{I}$	AC	0.234†	0.150	0.579	0.318	0.455
	SCC	0.122†	0.150	0.479	0.148	0.337
*K* ^Trans^ (mL/min/mL)	AC	0.479	0.056†	0.038	0.061	0.034
	SCC	0.239	0.039†	0.017	0.029	0.018

^†^ indicates marginally significant difference (*P *< 0.1) in the Wilcoxon rank sum test.

**Table 6:** Population mean of intratumoral median parameter values comparing AC (n = 5) and SCC (n = 5) NSCLC histologies with the five systemic arterial input tracer kinetic models.

Parameter	Histology	TK	ETK	2CX	AATH	DP
BF (mL/min/100g)	AC	258.9	30.80	109.5	73.61	64.75
	SCC	390.6	19.28	116.7	94.81	97.48
BV (mL/100g)	AC	36.86	11.14	24.32	18.37	22.39
	SCC	47.76	11.14	16.29	13.35	15.06
MTT (min)	AC	0.171	1.059	0.797	0.680	1.081
	SCC	0.193	2.405	1.232	0.937	1.344
PS (mL/min/100g)	AC	65.70	7.497	7.002	14.09	6.409*
	SCC	232.7	7.484	14.45	9.629	2.849*
$v_{I}$	AC	0.304†	0.151	0.474	0.228†	0.353*
	SCC	0.186†	0.150	0.234	0.107†	0.187*
*K* ^Trans^ (mL/min/mL)	AC	0.543	0.064	0.066	0.120	0.059*
	SCC	1.590	0.048	0.089	0.081	0.026*

“*” indicates statistically significant difference (*P* < 0.05) whereas “†” indicates marginally significant difference (*P* < 0.1) in the Wilcoxon rank sum test.

**Table 7:** Population mean of intratumoral median parameter values comparing AC (n = 5) and SCC (n = 5) NSCLC histologies with the five dual‐input tracer kinetic models.

Parameter	Histology	TK	ETK	2CX	AATH	DP
BF (mL/min/100g)	AC	226.8	26.26	107.8†	61.56	56.33
	SCC	127.1	16.71	51.10†	34.18	31.35
*γ*	AC	0.760	0.640	0.629	0.767	0.815
	SCC	0.759	0.468	0.826	0.916	0.907
BF_PA_ (mL/min/100g)	AC	113.1	17.43†	58.32	41.22†	41.48†
	SCC	60.96	6.720†	31.82	21.03†	21.31†
BF_A_ (mL/min/100g)	AC	42.00	7.372	27.16*	9.490	7.255
	SCC	21.84	8.911	4.118*	1.721	0.791
BV (mL/100g)	AC	38.11	11.05	22.16†	17.39†	19.68†
	SCC	24.48	12.07	10.23†	8.403†	9.516†
MTT (min)	AC	0.160	1.121†	0.870	0.798	1.309
	SCC	0.231	2.628†	1.641	1.373	1.944
PS (mL/min/100g)	AC	62.82	6.759	4.932†	8.015	4.110
	SCC	36.95	7.508	2.104†	3.803	2.209
$v_{I}$	AC	0.266†	0.151	0.472	0.238	0.461
	SCC	0.139†	0.152	0.404	0.143	0.344
*K* ^Trans^ (mL/min/mL)	AC	0.523	0.055	0.048†	0.069†	0.038
	SCC	0.297	0.042	0.020†	0.032†	0.019

“*” indicates statistically significant difference (*P* < 0.05) whereas “†” indicates marginally significant difference (*P* < 0.1) in the Wilcoxon rank sum test.

Pearson correlation between various parameters showed that, in MPM, BV was highly correlated between the 2CX and AATH models with pulmonary (*r* = 0.952), systemic (*r* = 0.964) or dual AIF (*r* = 0.954), respectively. In NSCLC, BF was highly correlated between the 2CX and AATH models with pulmonary (*r* = 0.954) or systemic AIF (*r* = 0.990), between the 2CX and DP models with systemic AIF (*r* = 0.979) and between the AATH and DP models with pulmonary (*r* = 0.969) or systemic AIF (*r* = 0.988), respectively. Blood volume was highly correlated between the 2CX and AATH models, between the 2CX and DP models and between the AATH and DP models with systemic AIF (*r* = 0.965, *r* = 0.961 and *r* = 0.968, respectively). Pulmonary arterial blood flow was highly correlated between the dual‐input AATH and DP models (*r* = 0.950).

**Figure 6:**
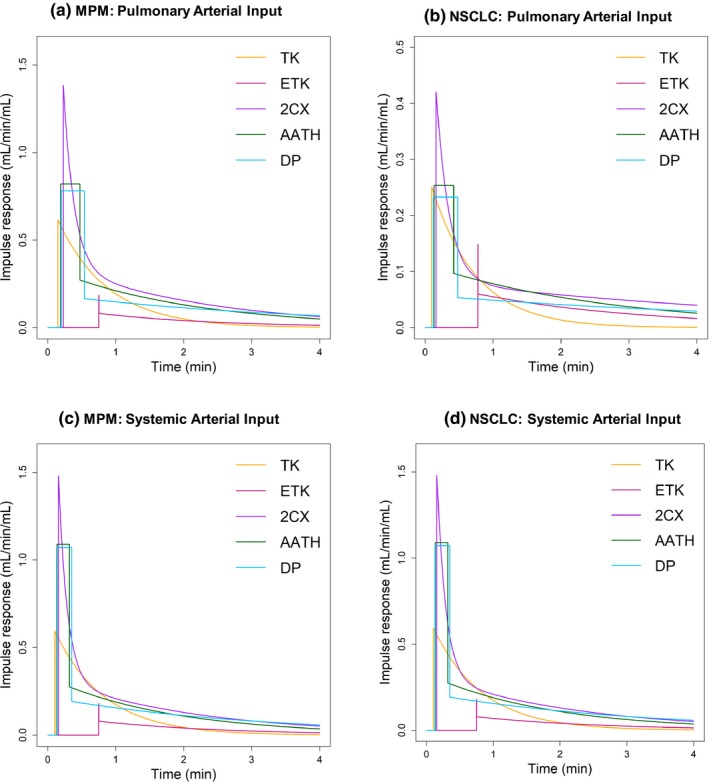
Impulse response function (IRF), i.e., $Q_{T}\left(t\right)$ derived from the mean values of the fitting parameters ($F/V_{P}$, $PS/V_{P}$, $v_{P}$, $v_{I}$, and $t_{\text{Lag},T}$) for each of the pulmonary arterial input Tofts‐Kety (TK), extended TK (ETK), two compartment exchange (2CX), adiabatic approximation to the tissue homogeneity (AATH), and distributed parameter (DP) models in (a) malignant pleural mesothelioma (MPM) and (b) nonsmall cell lung cancer (NSCLC). The IRF in (a) was derived from the mean values of the fitting parameters calculated from all voxels (n = 58,367) within the regions of the five MPM cases, whereas the IRF in (b) was derived from all voxels (n = 165,864) within those of the 10 NSCLC cases, respectively.

**Figure 7:**
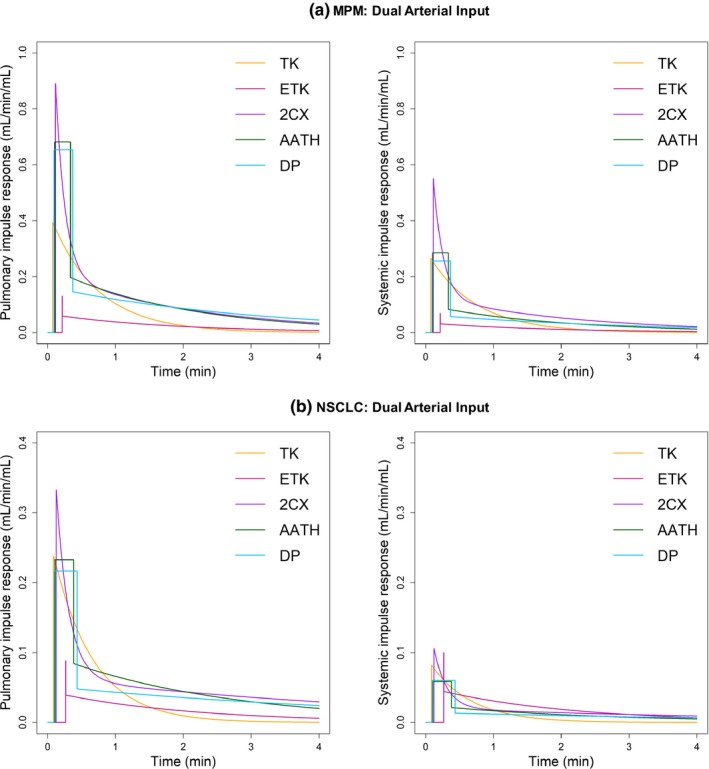
Pulmonary (left) and systemic (right) impulse response functions (IRFs), i.e., *Q*_T,PA_(*t*) and *Q*_T,A_(*t*) derived from the mean values of the fitting parameters ($F/V_{P}$, *γ*, $PS/V_{P}$, $v_{P}$, $v_{I}$, and $t_{\text{Lag},T}$) for each of the dual‐input Tofts‐Kety (TK), extended TK (ETK), two compartment exchange (2CX), adiabatic approximation to the tissue homogeneity (AATH), and distributed parameter (DP) models in malignant pleural mesothelioma (MPM) (upper) and nonsmall cell lung cancer (NSCLC) (lower). The upper IRFs in (a) and (b) were derived from the mean values of the fitting parameters calculated from all voxels (n = 58,367) within the regions of the five MPM cases, whereas the lower IRFs in (c) and (d) were derived from all voxels (n = 165,864) within those of the 10 NSCLC cases, respectively.

Figures 6 and 7 show the pulmonary and systemic IRFs derived from the mean values of the fitting parameters (i.e., $F/V_{P}$, $PS/V_{P}$, $v_{P}$, $v_{I}$ and $t_{\text{Lag},T}$ for single‐input models and along with an additional parameter *γ* for dual‐input models) for each of the five models in MPM and NSCLC. For the single‐input models, the peak of IRF represents perfusion ($F/V_{T}$) (Note that it is an extraction perfusion product for the TK model, i.e., $E\cdot F/V_{T}$). For the dual‐input models, the peaks of the pulmonary and systemic IRFs represent the pulmonary arterial perfusion ($F_{\text{PA}}/V_{T}$) and the systemic arterial perfusion ($F_{A}/V_{T}$), respectively (Note that they are extraction perfusion products for the TK model, i.e., $E\cdot F_{\text{PA}}/V_{T}$ and $E\cdot F_{A}/V_{T}$, respectively). In the pulmonary arterial input condition, $F/V_{T}$ was higher in MPM than in NSCLC, with all five models. In the systemic arterial input condition, $F/V_{T}$ was higher in MPM than in NSCLC, with all four models except for the 2CX model. In both types of tumors, $F_{\text{PA}}/V_{T}$ was higher than $F_{A}/V_{T}$, and both $F_{\text{PA}}/V_{T}$ and $F_{A}/V_{T}$ were higher in MPM than in NSCLC, with all four models except for the ETK model.

**Figure 8:**
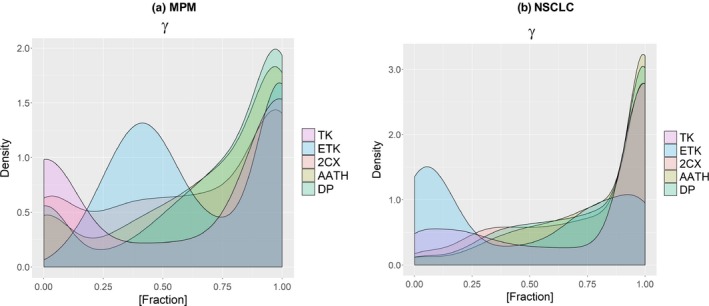
Kernel density estimation for the distribution of the pulmonary arterial flow fraction (*γ*) in (a) malignant pleural mesothelioma (MPM) and (b) nonsmall cell lung cancer (NSCLC). The distribution of γ values derived from the Tofts‐Kety (TK), two compartment exchange (2CX), adiabatic approximation to the tissue homogeneity (AATH), and distributed parameter (DP) models except for the extended TK (ETK) model tended to be left‐skewed in both types of tumors. The basic kernel estimator can be expressed as ${\hat{f}}_{\mathrm{kde}}\left(x\right)=\frac{1}{n}\sum_{i=1}^{n}K\left(\frac{x-x_{i}}{h}\right)$ *,* where* n* refers to independent observations *x_1_, x_2_, …, x_n_* from the random variable *X*, *K(·) is* the kernel function and* h* is the bandwidth. In this study, a Gaussian kernel with a random variable *X *= γ and a bandwidth* h* = 3 was used to produce smooth estimate.

In Figure 8, kernel density plots for the distribution of γ on all the voxels analyzed in MPM and NSCLC are shown. Kernel density plots were used to overcome the discreteness of the histogram by centering a smooth gaussian kernel function at each data point and then summing to get a density estimate. The distribution of *γ* values derived from all four models except for the ETK model tended to be left‐skewed (i.e., higher density of *γ* values close to unity than to zero) in both MPM and NSCLC, indicating that pulmonary arterial perfusion was higher than systemic arterial perfusion in both types of tumors. Even if the pulmonary arterial contribution to tumor perfusion was higher than the systemic arterial contribution in the TK, 2CX, AATH and DP models, the systemic arterial supply in those models would still be significant in both MPM and NSCLC.

**Figure 9:**
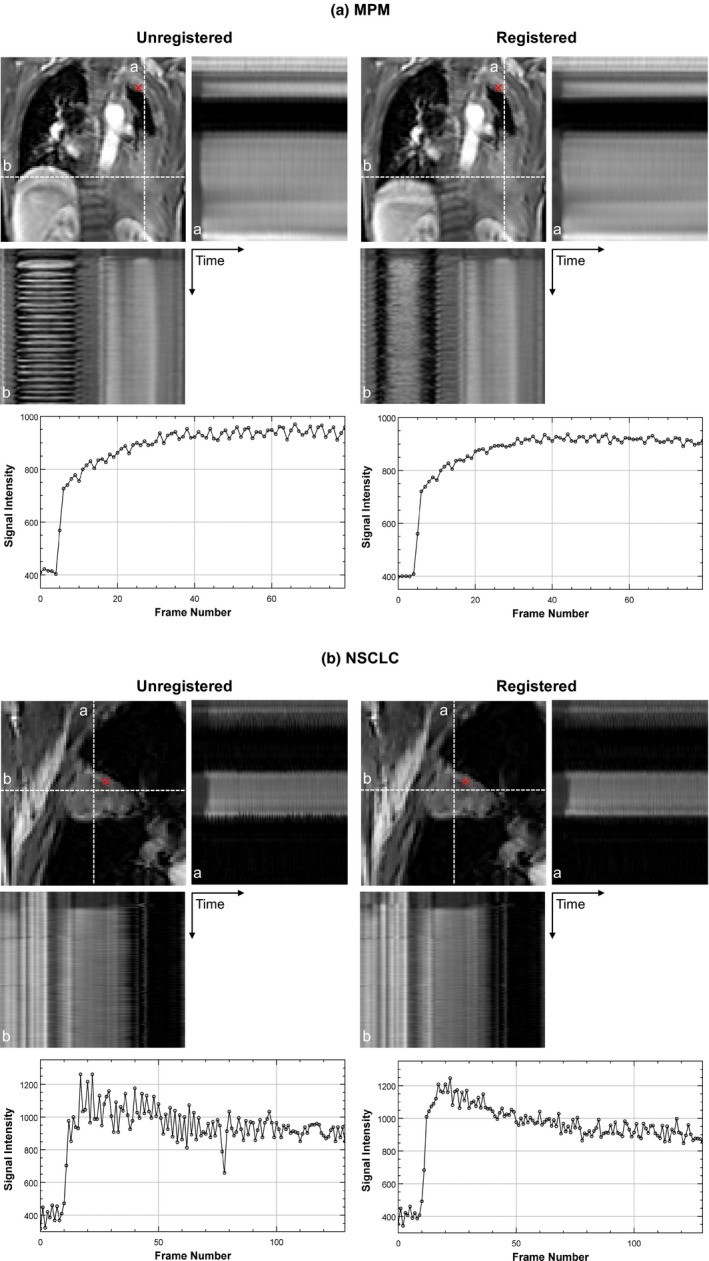
Effect of motion compensation for an example (top) MPM and (bottom) NSCLC case. Line profiles as a function of time along the superior‐inferior (a) and left‐right (b) direction are shown before and after registration. MR signal intensity curves at a point indicated by a red cross within tumor are also shown before and after registration. MPM, malignant pleural mesothelioma; NSCLC, nonsmall cell lung cancer.

Figure 9(a) and 9(b) shows the effect of motion compensation for an example MPM and NSCLC case. A line profile as a function of time along the left‐right and superior‐inferior direction is shown before and after registration. In addition, an MR signal intensity curve at a voxel sampled inside the tumor ROI is shown before and after registration. Large differences in kinetic parameter values were seen between registered and unregistered datasets for all the models. Table 8 shows the mean and standard deviation in the percent difference of parameter values obtained between registered and unregistered datasets of MPM and NSCLC patients for the dual‐input 2CX and AATH models. The large standard deviation in all the parameter values shows the importance of motion compensation of DCE‐MRI dataset.

**Table 8:** Mean and standard deviation in the percent difference of parameter values obtained between registered and unregistered dataset of MPM and NSCLC patients.

Parameter	MPM		NSCLC	
	2CX	AATH	2CX	AATH
BF (mL/min/100g)	−14 ± 60%	−20 ± 58%	12 ± 51%	20 ± 60%
*γ*	2 ± 17%	−5 ± 14%	2 ± 12%	−1 ± 12%
BF_PA_ (mL/min/100g)	−20 ± 46%	−12 ± 79%	26 ± 89%	19 ± 62%
BF_A_ (mL/min/100g)	−10 ± 85%	−19 ± 30%	5 ± 32%	30 ± 70%
BV (mL/100g)	−7 ± 44%	−5 ± 60%	−4 ± 15%	3 ± 22%
MTT (min)	24 ± 72%	10 ± 62%	17 ± 40%	3 ± 23%
PS (mL/min/100g)	−32 ± 47%	−35 ± 38%	−22 ± 31%	−14 ± 21%
$v_{I}$	−9 ± 21%	−16 ± 25%	16 ± 19%	5 ± 22%
*K* ^Trans^ (mL/min/mL)	−35 ± 45%	−33 ± 37%	−14 ± 27%	−5 ± 24%

MPM, malignant pleural mesothelioma; NSCLC, nonsmall cell lung cancer.

% difference = 100*(registered – unregistered)/unregistered.

## 4 DISCUSSION

Our pilot study showed that five different dual‐input tracer kinetic models with continuous‐time formalism can fit the DCE‐MRI data in MPM and NSCLC, to different degrees. The continuous‐time formalism enabled the realistic representation of arterial or tissue concentration‐time curves at any temporal resolution between data points, and thereby contributed to revealing realistic physiological characteristics in thoracic tumors that receive dual blood supply. The feasibility of dual‐input tracer kinetic modeling was confirmed by the results that it can fit the DCE‐MRI data better than single‐input tracer kinetic modeling, with the continuous‐time formalism. The proposed dual‐input models provide information about the relative contribution of the pulmonary and systemic arterial supplies to the tumor tissue from lung DCE‐MRI data. Our preliminary results also showed that the dual‐input tracer kinetic parameters have potential to differentiate tumor histologies in NSCLC. Knowledge of relative blood supply and quantification of tumor blood flow, capillary permeability and other microcirculatory parameters may have clinical implications in the management of thoracic malignancies 22, 43, 44.

Our results showed that the pulmonary arterial contribution to MPM is higher than the systemic arterial contribution with all four models except for the TK model (see Table 3). In a previous study using techniques to delineate pulmonary and bronchial vessels, Milne, et al. reported that the visceral pleural circulation is derived from and is continuous with the pulmonary circulation.^13^ The results from this study may be consistent with the above suggestion that MPM might derive a significant portion of its blood supply from the pulmonary circulation, though the blood supply to the visceral pleura is still controversial. A recent PCT study using the dual‐input maximum slope method also showed that dual blood supply of NSCLC is reflected by differences in perfusion parameters and is dependent on both tumor size and histological subtype.^26^ This PCT study showed that in general bronchial BF (i.e., BF_A_) was higher than pulmonary BF (i.e., BF_PA_) for NSCLC but noted that larger tumors had a higher pulmonary than bronchial contribution to their blood supply as compared with smaller tumors. The authors also observed higher mean BV for AC as compared to SCC. In our study, higher BV was also observed for AC with all four models except for the ETK model, but a discrepancy was found between their PCT and our DCE‐MRI studies in terms of higher BF_PA_ than BF_A_ for AC with all five models and for SCC with all four models except for the ETK model (Table 7). Further studies are necessary in order to conclude which kinetic model best approximates a true separation of pulmonary and bronchial perfusion for NSCLC, but it should be recognized that the maximum slope method systemically underestimates tissue perfusion due to the underlying assumption that there is no venous washout during the initial uptake phase.^45^ Estimates become progressively less accurate at higher flow values, at which the basic precondition of a negligible venous washout is violated because of the short capillary transit time ($V_{P}/F$) of CA molecules in the intravascular space, which might cause the discrepancy between their PCT and our DCE‐MRI studies.

PCT studies have used the maximum slope method for dual‐input perfusion analysis. PCT uses a short acquisition time (30–60 s) with high temporal resolution (0.2–2 s) to keep an acceptable radiation dose in PCT protocol as well as to separate the peak of time density curves of the pulmonary and systemic arteries.^46^, ^47^ By contrast, DCE‐MRI acquisitions are made over a few minutes mainly due to their nonionizing radiation nature, enabling tracer kinetic analysis in two tissue compartments. This study, with more complex models, involves the fitting of more kinetic parameters that describe CA exchange behavior between the capillary plasma and interstitial spaces and the fractional volumes of the CA in the two compartments in addition to tissue perfusion. Therefore, our proposed method can provide a more comprehensive assessment of microvascular physiologic properties for thoracic malignancies as well as more accurately quantify pulmonary perfusion, and could be of clinical value for disease evaluation and assessment of treatment response.

The cAIC_min_ maps showed a heterogeneous pattern with spatially contiguous regions in which one of the models outperformed the others. This indicates that the underlying properties of the tumor microvasculature and microenvironment are heterogeneous. Because the TK and ETK models provided a relatively poor fit to the data as compared with the other three models, their *w_m_* were low (<0.25) in the majority of voxels, showing highly right‐skewed distributions. The *w_m_* for the AATH and DP models were relatively evenly distributed, as compared with those for the TK and ETK models, but for the most part ranged between 0 and 0.5. The 2CX model was most evenly distributed across the range of *w_m_* and outperformed other models in the range of *w_m_* > 0.75 in both types of tumors. Pearson correlation analysis showed that BV was consistently highly correlated between the 2CX and AATH models in MPM with single or dual AIF (*r*> 0.95). In NSCLC, BF_PA_ was highly correlated between the AATH and DP models (*r* = 0.95), but no correlations were higher than *r* = 0.7 between BF_A_ estimated using the five models, indicating that BF_A_ is relatively more different than BF_PA_ between different models.

The results showe that the dual‐input 2CX model gave the best fit among all single‐ and dual‐input kinetic models in the voxel‐based analysis for MPM and NSCLC tumors (Table 4). In a previous DCE‐MRI study of lung tumors, Naish, et al. have shown that the AATH model, in most cases, gave the best description of the data.^21^ The study compared three different models (i.e., TK, ETK and AATH models) based on a single pulmonary AIF using DCE‐MRI data with 4‐s temporal resolution. The fact that the AATH was the best‐fit model is consistent with our results for MPM with the pulmonary AIF (Tables 1 and 4), although our results for NSCLC with the pulmonary AIF showed that 2CX was the best‐fit model. If a compartment is observed at time intervals much longer than the time required for the mixing of CA within the compartment, it would present the appearance of a well‐mixed compartment.^48^ For instance, if the CA traverses the capillary plasma space in tissue for a short time, and the time interval between dynamic scans is much longer, then the 2CX model would be more appropriate than the AATH model. By contrast, if the time interval between dynamic scans is comparable to the capillary transit time ($V_{P}/F$) of the CA in the tissue, then the plasma compartment may not appear to be homogeneous and the AATH model would be appropriate. The applicability of a particular model would not only rely on the computational approach based on underlying tissue physiology, but also the imaging protocol^49^ and noise condition in the data.^50^ From the results, it is observed that the frequency for the dual‐input AATH model to have cAIC_min_ was 4.3% higher in NSCLC (35.4%) than in MPM (31.1%). When it is considered that the DCE‐MRI data for NSCLC had higher temporal resolution (2 s) than those for MPM (5 s), better performance of the AATH model in NSCLC is plausible. It should also be recognized that the computational method used in this study, which is based on the explicit model solution in the continuous‐time domain, would minimize the effect of different temporal resolutions in the data when tissue microvascular parameters are estimated.^51^

To the best of our knowledge, there have been no studies done on the comparisons among various tracer kinetic models with single or dual arterial input condition. We showed that the 2CX model could describe the DCE‐MRI data better than other models across all three arterial input conditions, and that the dual‐input 2CX was the best‐fit model in thoracic tumors. The dual‐input 2CX model‐derived BF_A_ was most statistically significant and the dual‐input 2CX model‐derived BF, BV, PS and *K* ^Trans^ were marginally significant in differentiating between AC and SCC, which were all higher in AC than in SCC. Although there have been no comparable DCE‐MRI studies for distinguishing between AC and SCC, PCT studies concerning the perfusion characterization of AC and SCC have reported consistent results with equivalent parameters. A previous study showed that BV and flow‐extraction product (i.e., *K* ^Trans^ in this study) are significantly higher in AC than in SCC.^52^ Another study also showed that before treatments, the AC histological type has a BF mean value significantly greater than SCC subtype.^53^ Even though the systemic arterial input DP model‐derived PS, $v_{I}$ and *K* ^Trans^ appeared statistically significant, the fitting error of the systemic arterial input DP model was considerably larger than that of the dual‐input 2CX model in NSCLC (Table 4).

There are some limitations in our study. The number of patients investigated in this study is small, although it serves as a proof‐of‐concept comparative study. Although the preliminary analysis is encouraging, a large patient cohort analysis is needed to further validate the clinical applicability of dual‐input tracer kinetic analysis approach in thoracic malignancies. We would also like to point out that a good fit may not necessarily imply accurate parameter estimates because an improved fit could be due to the sparsity of the data, rather than the accuracy of the model for describing tissue kinetics.^54^ Therefore, a model selection criterion based on the goodness‐of‐fit has limited value, though it allows for finding a best‐fit model for the unknown true data given in the experimental protocol. Even if the dual‐input 2CX model was the best‐fit model in this study, the underlying physiologic assumption of the 2CX model that the administered CA is instantaneously mixed in capillaries might be unrealistic. Finally, the appropriateness of an AIF model would depend on the CA injection protocol, temporal resolution and imaging duration, etc., though the proposed AIF model was flexible enough to fit the individual (patient‐specific) AIFs in this study.

## 5 CONCLUSION

In this study, five different single‐ or dual‐input tracer kinetic models were compared with respect to goodness‐of‐fit statistics using cAIC in the analysis of DCE‐MRI data for MPM and NSCLC. For most voxels, the dual‐input 2CX model was best in describing the CA concentration of the two thoracic malignancies. Potential clinical value of the dual‐input tracer kinetic modeling was demonstrated by comparing parameter values between two NSCLC histologic subtypes, i.e., AC and SCC, in which the 2CX‐model‐derived BF_A_ in AC was significantly higher than that in SCC. The choice of a model influenced the contribution of pulmonary versus systemic arterial flow to the total pulmonary perfusion in the tumors. Although the pulmonary arterial flow was higher than the systemic arterial flow in both MPM and NSCLC, the systemic arterial contribution to tumor perfusion could still be substantial and may impact the prognostic and predictive value of DCE‐MRI metrics for thoracic malignancies.

## CONFLICT OF INTEREST

None.

## Supporting information


**SupInfo S1.** Supplementary document 1.

**SupInfo S2.** Supplementary document 2.
