# Supplementary material for: Dual‐input tracer kinetic modeling of dynamic contrast‐enhanced MRI in thoracic malignancies
Source: J Appl Clin Med Phys. 2019 Oct 11;20(11):169–88. doi: 10.1002/acm2.12740 (PMC6839367; doi:10.1002/acm2.12740)
Supplement: Supplementary file 1 — SupInfo S1. Supplementary document 1. [file ACM2-20-169-s001.pdf]

Supplementary document 1

A. Tissue residue functions of the five tracer kinetic models

Tracer transport within the capillary-tissue system can be modeled as a linear, time-invariant (stationary) system. All models considered in this study fall under this assumption. Each model represents a TRF, $R_{\mathrm{T}}(t)$, which can be modeled by considering tracer kinetics for capillary-tissue exchange in simplified or the full two compartments: the plasma space and the interstitial space.

The 2CX model assumes well-mixed (homogeneous) compartments so that CA concentration is a function of only time, not space. The TRF for the 2CX model is a bi-exponential function of the form:

$$R_{\mathrm{T,2CX}}(t) = \left[Ae^{\alpha t} + (1-A)e^{\beta t}\right]u(t)$$
$$\text{with } \binom{\alpha}{\beta} = \frac{1}{2}\left[-\left\{\frac{F}{V_{\mathrm{P}}} + \left(1+\frac{v_{\mathrm{P}}}{v_{\mathrm{I}}}\right)\frac{PS}{V_{\mathrm{P}}}\right\} \pm \sqrt{\left\{\frac{F}{V_{\mathrm{P}}} + \left(1+\frac{v_{\mathrm{P}}}{v_{\mathrm{I}}}\right)\frac{PS}{V_{\mathrm{P}}}\right\}^2 - 4\frac{v_{\mathrm{P}}}{v_{\mathrm{I}}}\frac{F}{V_{\mathrm{P}}}\frac{PS}{V_{\mathrm{P}}}}\right] \tag{1}$$
$$\text{and } A = \frac{\alpha + \left(1+\frac{v_{\mathrm{P}}}{v_{\mathrm{I}}}\right)\frac{PS}{V_{\mathrm{P}}}}{\alpha - \beta},$$

where v_{P} and v_{I} are the relative volumes of the plasma and interstitial spaces in the considered tissue region with volume V_{T}, PS is the CA-specific permeability surface area product (in mL/min), V_{P} (in mL) is the plasma volume, and $u(t)$ is the unit step function that explains $R_{\mathrm{T}}(t)$ is valid only at $t \geq 0$.

The AATH model describes a closed-form solution of the Johnson and Wilson tissue homogeneity model in the time domain by considering adiabatic (slow) changes of CA concentration in the interstitial compartment relative to that in the plasma compartment [1], whereby this model accounts for the concentration gradient of CA between the arterial and venous ends of the capillary, but CA concentration in the interstitial space is assumed to be well mixed. The TRF for the AATH model is given by:

$$R_{\mathrm{T,AATH}}(t) = u(t) + \left[Ee^{-\frac{v_{\mathrm{P}}EF}{v_{\mathrm{I}}V_{\mathrm{P}}}\left(t-\frac{V_{\mathrm{P}}}{F}\right)} - 1\right]u\left(t-\frac{V_{\mathrm{P}}}{F}\right), \tag{2}$$

where $E = 1 - e^{-PS/F}$ is the first-pass extraction fraction from the plasma space to the interstitial space, and V_{P}/F (in min) is the capillary transit time.

The DP model also describes the concentration gradient of CA along the axial length of a capillary tube like the AATH model, but unlike the AATH model, the interstitial compartment is modeled as a series of infinitesimal compartments that exchange CA only with nearby locations in the capillary bed. Thus, the CA concentrations in the plasma and interstitial compartments both depend on the position of the capillary tube. The TRF for the DP model is given by:

$$R_{\mathrm{T,DP}}(t) = u(t) - e^{-\frac{PS}{F}}\left[1 + \frac{PS}{V_{\mathrm{P}}}\int_0^{t-\frac{V_{\mathrm{P}}}{F}} e^{-\frac{v_{\mathrm{P}}PS}{v_{\mathrm{I}}V_{\mathrm{P}}}\tau}\sqrt{\frac{v_{\mathrm{P}}}{v_{\mathrm{I}}}\frac{V_{\mathrm{P}}}{F}\frac{1}{\tau}}\, I_1\left(2\frac{PS}{V_{\mathrm{P}}}\sqrt{\frac{v_{\mathrm{P}}}{v_{\mathrm{I}}}\frac{V_{\mathrm{P}}}{F}\tau}\right)d\tau\right]u\left(t - \frac{V_{\mathrm{P}}}{F}\right), \quad (3)$$

where $I_1(t)$ denotes the modified Bessel function of the first kind. The integral term, including the modified Bessel function, cannot be solved into a fully analytic form. To simplify the formulation of the integral term in Equation A3, an alternative derivation can be considered by evaluating a Taylor series expansion [2]. The $R_{\mathrm{T,DP}}(t)$ can be simplified as:

$$R_{\mathrm{T,DP}}(t) \cong u(t) - e^{-\frac{PS}{F}}\left[1 + \frac{v_{\mathrm{P}}}{v_{\mathrm{I}}}\frac{PS}{V_{\mathrm{P}}}\frac{PS}{F}\left(t - \frac{V_{\mathrm{P}}}{F}\right)\right]u\left(t - \frac{V_{\mathrm{P}}}{F}\right). \quad (4)$$

The ETK model assumes negligible capillary transit time in comparison to the data sampling interval, resulting in a situation where CA plasma concentration is equal to the AIF. The TRF for the ETK model is given by

$$R_{\mathrm{T,ETK}}(t) = \frac{V_{\mathrm{P}}}{F}\delta(t) - Ee^{-\frac{v_{\mathrm{P}}EF}{v_{\mathrm{I}}V_{\mathrm{P}}}t}u(t), \quad (5)$$

where $\delta(t)$ is the Dirac delta function that denotes the idealized impulse excitation of a unit mass source at $t = 0$. The TK model provides a further simplification of the two-compartment situation, whereby it is assumed that $v_{\mathrm{P}} \ll v_{\mathrm{I}}$ and thus the contribution of CA in the plasma compartment to $C_{\mathrm{T}}(t)$ is ignored. The TRF for the TK model is given by

$$R_{\mathrm{T,TK}}(t) = Ee^{-\frac{v_{\mathrm{P}}EF}{v_{\mathrm{I}}V_{\mathrm{P}}}t}u(t). \quad (6)$$

The symbols and definitions for kinetic parameters used in the current study are summarized in supplementary document 2.

B. Modeling of the dual-input function

Because the dual arterial inputs join in the capillary bed, they can be effectively replaced by a single net input function $C_{\mathrm{in}}(t)$ with their mixed contributions, i.e., a weighted sum of the pulmonary and systemic AIFs: $C_{\mathrm{in}}(t) = \gamma C_{\mathrm{PA}}(t) + (1-\gamma)C_{\mathrm{A}}(t)$. The dual-input functions can be further decomposed into $C_{\mathrm{PA}}(t) = C_{\mathrm{B,PA}}(t) + C_{\mathrm{B,PA}}(t) \otimes G_{\mathrm{PA}}(t)$ and $C_{\mathrm{A}}(t) = C_{\mathrm{B,A}}(t) + C_{\mathrm{B,A}}(t) \otimes G_{\mathrm{A}}(t)$, respectively, where $C_{\mathrm{B,PA}}(t) = a_{\mathrm{B,PA}} t e^{-\mu_{\mathrm{B,PA}} t} u(t)$ and $C_{\mathrm{B,A}}(t) = a_{\mathrm{B,A}} t e^{-\mu_{\mathrm{B,A}} t} u(t)$ are the bolus function that describes the first-pass of the bolus of CA for the pulmonary and systemic arterial components, and $G_{\mathrm{PA}}(t) = a_{\mathrm{G,PA}} e^{-\mu_{\mathrm{G,PA}} t} u(t)$ and $G_{\mathrm{A}}(t) = a_{\mathrm{G,A}} e^{-\mu_{\mathrm{G,A}} t} u(t)$ are the body transfer function (BTF) that models leakage into the whole-body interstitial space during the recirculation phase. Thus, $C_{\mathrm{PA}}(t)$ and $C_{\mathrm{A}}(t)$ are modeled as a full-pass AIF that describes superposition of the bolus shape (first-pass) and its shape after modification by the BTF (recirculation). An AIF model that represents a sums-of-exponentials function can be adopted for modeling the dual-input arterial curves as follows [3]:

$$C_{\mathrm{PA}}(t) = \left[A_{\mathrm{B,PA}} t e^{-\mu_{\mathrm{B,PA}} t} + A_{\mathrm{G,PA}}\left(e^{-\mu_{\mathrm{G,PA}} t} - e^{-\mu_{\mathrm{B,PA}} t}\right)\right] u(t), \tag{7}$$

$$C_{\mathrm{A}}(t) = \left[A_{\mathrm{B,A}} t e^{-\mu_{\mathrm{B,PA}} t} + A_{\mathrm{G,A}}\left(e^{-\mu_{\mathrm{G,A}} t} - e^{-\mu_{\mathrm{B,A}} t}\right)\right] u(t), \tag{8}$$

where $A_{\mathrm{B,PA}} = a_{\mathrm{B,PA}} - a_{\mathrm{B,PA}} a_{\mathrm{G,PA}} / \left(\mu_{\mathrm{B,PA}} - \mu_{\mathrm{G,PA}}\right)$, $A_{\mathrm{G,PA}} = a_{\mathrm{B,PA}} a_{\mathrm{G,PA}} / \left(\mu_{\mathrm{B,PA}} - \mu_{\mathrm{G,PA}}\right)^2$, $\mu_{\mathrm{B,PA}}$ and $\mu_{\mathrm{G,PA}}$ are scaling constants which govern the height and shape of the pulmonary AIF, and $A_{\mathrm{B,A}} = a_{\mathrm{B,A}} - a_{\mathrm{B,A}} a_{\mathrm{G,A}} / \left(\mu_{\mathrm{B,A}} - \mu_{\mathrm{G,A}}\right)$, $A_{\mathrm{G,A}} = a_{\mathrm{B,A}} a_{\mathrm{G,A}} / \left(\mu_{\mathrm{B,A}} - \mu_{\mathrm{G,A}}\right)^2$, $\mu_{\mathrm{B,A}}$ and $\mu_{\mathrm{G,A}}$ are those of the systemic AIF. By imposing the time lag of the first-pass bolus arrival to the pulmonary artery and the aorta ($t_{\mathrm{Lag,PA1}}$ and $t_{\mathrm{Lag,A1}}$), and the difference in onset times of the first-pass and the recirculation ($t_{\mathrm{Lag,PA2}}$ and $t_{\mathrm{Lag,A2}}$) on Equations (A1) and (A2), respectively, $C_{\mathrm{PA}}(t)$ and $C_{\mathrm{A}}(t)$ take the forms

$$C_{\mathrm{PA}}(t) = C_{\mathrm{B,PA}}\left(t - t_{\mathrm{Lag,PA1}}\right) + C_{\mathrm{B,PA}}\left(t - t_{\mathrm{Lag,PA1}} - t_{\mathrm{Lag,PA2}}\right) \otimes G_{\mathrm{PA}}(t), \tag{9}$$

$$C_{\mathrm{A}}(t) = a_{\mathrm{B,A}}\left(t - t_{\mathrm{Lag,A1}}\right) e^{-\mu_{\mathrm{B,A}}\left(t - t_{\mathrm{Lag,A1}}\right)} u\left(t - t_{\mathrm{Lag,A1}}\right) - \frac{a_{\mathrm{B,A}} a_{\mathrm{G,A}}}{\mu_{\mathrm{B,A}} - \mu_{\mathrm{G,A}}} \left[\left(t - t_{\mathrm{Lag,A1}} - t_{\mathrm{Lag,A2}}\right) e^{-\mu_{\mathrm{B,A}}\left(t - t_{\mathrm{Lag,A1}} - t_{\mathrm{Lag,A2}}\right)} - \frac{e^{-\mu_{\mathrm{G,A}}\left(t - t_{\mathrm{Lag,A1}} - t_{\mathrm{Lag,A2}}\right)} - e^{-\mu_{\mathrm{B,A}}\left(t - t_{\mathrm{Lag,A1}} - t_{\mathrm{Lag,A2}}\right)}}{\mu_{\mathrm{B,A}} - \mu_{\mathrm{G,A}}}\right] u\left(t - t_{\mathrm{Lag,A1}} - t_{\mathrm{Lag,A2}}\right), \tag{10}$$

where $C_{\mathrm{PA}}(t)$ was expressed with its convolution components for providing abbreviated reference, while $C_{\mathrm{A}}(t)$ with its analytic solution. Note that the analytic solution of $C_{\mathrm{PA}}(t)$ is of the same form as that of $C_{\mathrm{A}}(t)$, though the scaling constants and the onset times should be distinguished between them.

C. Analytic solutions of the five different models

For convenience in expressing analytic solutions of $C_{\mathrm{T}}(t)$ for each model, it was defined that $C_{\mathrm{T,PA}}(t) = Q_{\mathrm{T}}(t - t_{\mathrm{Lag,T}}) \otimes C_{\mathrm{PA}}(t)/(1 - H_{\mathrm{LV}})$, and $C_{\mathrm{T,A}}(t) = Q_{\mathrm{T}}(t - t_{\mathrm{Lag,T}}) \otimes C_{\mathrm{A}}(t)/(1 - H_{\mathrm{LV}})$. Once the dual AIF is modeled as a continuous-time parametric functional form, a subsequent analytic solution of $C_{\mathrm{T}}(t)$ can be derived by incorporating the scaling constants and the onset times of the dual AIF into the convolution integral with the TRF for each tracer kinetic model. By assuming that tracer transport within the capillary-tissue system can be modeled as a linear time-invariant (stationary) system, the analytic form of $C_{\mathrm{T}}(t)$ can be given by

$$C_{\mathrm{T}}(t) = \gamma C_{\mathrm{T,PA}}(t) + (1-\gamma) C_{\mathrm{T,A}}(t) = \frac{F}{V_{\mathrm{T}}} \frac{R_{\mathrm{T}}(t)}{1-H_{\mathrm{LV}}} \otimes \left[\gamma\left\{C_{\mathrm{B,PA}}(t_{\mathrm{PA1}}) + C_{\mathrm{B,PA}}(t_{\mathrm{PA2}}) \otimes G_{\mathrm{PA}}(t)\right\} + (1-\gamma)\left\{C_{\mathrm{B,A}}(t_{\mathrm{A1}}) + C_{\mathrm{B,A}}(t_{\mathrm{A2}}) \otimes G_{\mathrm{A}}(t)\right\}\right], \tag{11}$$

where $t_{\mathrm{PA1}} = t - t_{\mathrm{Lag,PA1}} - t_{\mathrm{Lag,T}}$, $t_{\mathrm{PA2}} = t_{\mathrm{PA1}} - t_{\mathrm{Lag,PA2}}$, $C_{\mathrm{T,PA}}(t) = \frac{F}{V_{\mathrm{T}}} \frac{R_{\mathrm{T}}(t)}{1-H_{\mathrm{LV}}} \otimes \left[C_{\mathrm{B,PA}}(t_{\mathrm{PA1}}) + C_{\mathrm{B,PA}}(t_{\mathrm{PA2}}) \otimes G_{\mathrm{PA}}(t)\right]$, $t_{\mathrm{A1}} = t - t_{\mathrm{Lag,A1}} - t_{\mathrm{Lag,T}}$, $t_{\mathrm{A2}} = t_{\mathrm{A1}} - t_{\mathrm{Lag,A2}}$, and $C_{\mathrm{T,A}}(t) = \frac{F}{V_{\mathrm{T}}} \frac{R_{\mathrm{T}}(t)}{1-H_{\mathrm{LV}}} \otimes \left[C_{\mathrm{B,A}}(t_{\mathrm{A1}}) + C_{\mathrm{B,A}}(t_{\mathrm{A2}}) \otimes G_{\mathrm{A}}(t)\right]$.

The analytic solutions of $C_{\mathrm{T,A}}(t)$ for the five different tracer kinetic models are given explicitly in the continuous-time domain by

$$C_{\mathrm{T,A,2CX}}(t) = \frac{F}{V_{\mathrm{T}}} \frac{1}{1-H_{\mathrm{LV}}} \left[\left\{K_{\mathrm{A1}} e^{\alpha t_{\mathrm{A1}}} + K_{\mathrm{A2}} e^{\beta t_{\mathrm{A1}}} + K_{\mathrm{A3}} e^{-\mu_{\mathrm{B,A}} t_{\mathrm{A1}}} + K_{\mathrm{A4}} t_{\mathrm{A1}} e^{-\mu_{\mathrm{B,A}} t_{\mathrm{A1}}}\right\} u(t_{\mathrm{A1}}) + \left\{K_{\mathrm{A5}} e^{\alpha t_{\mathrm{A2}}} + K_{\mathrm{A6}} e^{\beta t_{\mathrm{A2}}} + K_{\mathrm{A7}} e^{-\mu_{\mathrm{B,A}} t_{\mathrm{A2}}} + K_{\mathrm{A8}} e^{-\mu_{\mathrm{G,A}} t_{\mathrm{A2}}} + K_{\mathrm{A9}} t_{\mathrm{A2}} e^{-\mu_{\mathrm{B,A}} t_{\mathrm{A2}}}\right\} u(t_{\mathrm{A2}})\right], \tag{12}$$

where $K_{\mathrm{A1}} = \frac{A a_{\mathrm{B,A}}}{(\alpha+\mu_{\mathrm{B,A}})^2}$, $K_{\mathrm{A2}} = \frac{(1-A) a_{\mathrm{B,A}}}{(\beta+\mu_{\mathrm{B,A}})^2}$, $K_{\mathrm{A3}} = -(K_{\mathrm{A1}} + K_{\mathrm{A2}})$, $K_{\mathrm{A4}} = -\left\{K_{\mathrm{A1}}(\alpha + \mu_{\mathrm{B,A}}) + K_{\mathrm{A2}}(\beta + \mu_{\mathrm{B,A}})\right\}$, $K_{\mathrm{A5}} = \frac{K_{\mathrm{A1}} a_{\mathrm{G,A}}}{\alpha+\mu_{\mathrm{G,A}}}$, $K_{\mathrm{A6}} = \frac{K_{\mathrm{A2}} a_{\mathrm{G,A}}}{\beta+\mu_{\mathrm{G,A}}}$, $K_{\mathrm{A7}} = \frac{a_{\mathrm{G,A}}\left\{K_{\mathrm{A1}}(\alpha+2\mu_{\mathrm{B,A}}-\mu_{\mathrm{G,A}}) + K_{\mathrm{A2}}(\beta+2\mu_{\mathrm{B,A}}-\mu_{\mathrm{G,A}})\right\}}{(\mu_{\mathrm{B,A}}-\mu_{\mathrm{G,A}})^2}$, $K_{\mathrm{A8}} = -\frac{a_{\mathrm{B,A}}\left\{A\frac{K_{\mathrm{A5}}}{K_{\mathrm{A1}}} + (1-A)\frac{K_{\mathrm{A6}}}{K_{\mathrm{A2}}}\right\}}{(\mu_{\mathrm{B,A}}-\mu_{\mathrm{G,A}})^2}$, and $K_{\mathrm{A9}} = -\frac{K_{\mathrm{A4}} a_{\mathrm{G,A}}}{\mu_{\mathrm{B,A}}-\mu_{\mathrm{G,A}}}$,

$$C_{\mathrm{T,A,AATH}}(t) = \frac{F}{V_{\mathrm{T}}}\frac{1}{1-H_{\mathrm{LV}}}\Bigg[\{L_{\mathrm{A1}}(1-e^{-\mu_{\mathrm{B,A}}t_{\mathrm{A1}}}) + L_{\mathrm{A2}}t_{\mathrm{A1}}e^{-\mu_{\mathrm{B,A}}t_{\mathrm{A1}}}\}u(t_{\mathrm{A1}}) + \Big\{L_{\mathrm{A3}} + L_{\mathrm{A4}}e^{-\mu_{\mathrm{B,A}}t_{\mathrm{A2}}} + L_{\mathrm{A5}}e^{-\mu_{\mathrm{G,A}}t_{\mathrm{A2}}} - \frac{L_{\mathrm{A2}}a_{\mathrm{G,A}}}{\mu_{\mathrm{B,A}}-\mu_{\mathrm{G,A}}}t_{\mathrm{A2}}e^{-\mu_{\mathrm{B,A}}t_{\mathrm{A2}}}\Big\}u(t_{\mathrm{A2}}) + \Big\{L_{\mathrm{A6}}e^{-L_1\left(t_{\mathrm{A1}}-\frac{V_{\mathrm{P}}}{F}\right)} - L_{\mathrm{A1}} + L_{\mathrm{A7}}e^{-\mu_{\mathrm{B,A}}\left(t_{\mathrm{A1}}-\frac{V_{\mathrm{P}}}{F}\right)} + L_{\mathrm{A8}}\left(t_{\mathrm{A1}}-\frac{V_{\mathrm{P}}}{F}\right)e^{-\mu_{\mathrm{B,A}}\left(t_{\mathrm{A1}}-\frac{V_{\mathrm{P}}}{F}\right)}\Big\}u\left(t_{\mathrm{A1}}-\frac{V_{\mathrm{P}}}{F}\right) + \Big\{L_{\mathrm{A9}}e^{-L_1\left(t_{\mathrm{A2}}-\frac{V_{\mathrm{P}}}{F}\right)} - L_{\mathrm{A3}} + L_{\mathrm{A10}}e^{-\mu_{\mathrm{B,A}}\left(t_{\mathrm{A2}}-\frac{V_{\mathrm{P}}}{F}\right)} + L_{\mathrm{A11}}e^{-\mu_{\mathrm{G,A}}\left(t_{\mathrm{A2}}-\frac{V_{\mathrm{P}}}{F}\right)} + L_{\mathrm{A12}}\left(t_{\mathrm{A2}}-\frac{V_{\mathrm{P}}}{F}\right)e^{-\mu_{\mathrm{B,A}}\left(t_{\mathrm{A2}}-\frac{V_{\mathrm{P}}}{F}\right)}\Big\}u\left(t_{\mathrm{A2}}-\frac{V_{\mathrm{P}}}{F}\right)\Bigg], \tag{13}$$

where $L_{\mathrm{A1}} = \frac{a_{\mathrm{B,A}}}{\mu_{\mathrm{B,A}}{}^2}$, $L_{\mathrm{A2}} = -L_{\mathrm{A1}}\mu_{\mathrm{B,A}}$, $L_{\mathrm{A3}} = \frac{L_{\mathrm{A1}}a_{\mathrm{G,A}}}{\mu_{\mathrm{G,A}}}$, $L_{\mathrm{A4}} = \frac{a_{\mathrm{G,A}}}{\mu_{\mathrm{B,A}}-\mu_{\mathrm{G,A}}}\left(L_{\mathrm{A1}} - \frac{L_{\mathrm{A2}}}{\mu_{\mathrm{B,A}}-\mu_{\mathrm{G,A}}}\right)$, $L_{\mathrm{A5}} = -\frac{a_{\mathrm{B,A}}a_{\mathrm{G,A}}}{\mu_{\mathrm{G,A}}(\mu_{\mathrm{B,A}}-\mu_{\mathrm{G,A}})^2}$, $L_{\mathrm{A6}} = \frac{Ea_{\mathrm{B,A}}}{(\mu_{\mathrm{B,A}}-L_1)^2}$, $L_{\mathrm{A7}} = L_{\mathrm{A1}} - L_{\mathrm{A6}}$, $L_{\mathrm{A8}} = L_{\mathrm{A1}}\mu_{\mathrm{B,A}} - L_{\mathrm{A6}}(\mu_{\mathrm{B,A}} - L_1)$, $L_{\mathrm{A9}} = \frac{L_{\mathrm{A6}}a_{\mathrm{G,A}}}{\mu_{\mathrm{G,A}}-L_1}$, $L_{\mathrm{A10}} = \frac{L_{\mathrm{A6}}a_{\mathrm{G,A}}}{\mu_{\mathrm{B,A}}-\mu_{\mathrm{G,A}}}\left(1 + \frac{\mu_{\mathrm{B,A}}-L_1}{\mu_{\mathrm{B,A}}-\mu_{\mathrm{G,A}}}\right) - L_{\mathrm{A4}}$, $L_{\mathrm{A11}} = -\frac{L_{\mathrm{A2}}\mu_{\mathrm{B,A}}a_{\mathrm{G,A}}}{(\mu_{\mathrm{B,A}}-\mu_{\mathrm{G,A}})^2}\left(\frac{1}{\mu_{\mathrm{G,A}}} - \frac{E}{\mu_{\mathrm{G,A}}-L_1}\right)$, and $L_{\mathrm{A12}} = \frac{L_{\mathrm{A2}}\mu_{\mathrm{B,A}}a_{\mathrm{G,A}}}{\mu_{\mathrm{B,A}}-\mu_{\mathrm{G,A}}}\left(\frac{1}{\mu_{\mathrm{B,A}}} - \frac{E}{\mu_{\mathrm{B,A}}-L_1}\right)$, where $L_1 = \frac{v_{\mathrm{P}}}{v_{\mathrm{I}}}\frac{EF}{V_{\mathrm{P}}}$,

$$C_{\mathrm{T,A,DP}}(t) = \frac{F}{V_{\mathrm{T}}}\frac{1}{1-H_{\mathrm{LV}}}\Bigg[\{M_{\mathrm{A1}}(1-e^{-\mu_{\mathrm{B,A}}t_{\mathrm{A1}}}) + M_{\mathrm{A2}}t_{\mathrm{A1}}e^{-\mu_{\mathrm{B,A}}t_{\mathrm{A1}}}\}u(t_{\mathrm{A1}}) + \{M_{\mathrm{A3}} + M_{\mathrm{A4}}e^{-\mu_{\mathrm{B,A}}t_{\mathrm{A2}}} + M_{\mathrm{A5}}e^{-\mu_{\mathrm{G,A}}t_{\mathrm{A2}}} + M_{\mathrm{A6}}t_{\mathrm{A2}}e^{-\mu_{\mathrm{B,A}}t_{\mathrm{A2}}}\}u(t_{\mathrm{A2}}) + \Big\{M_{\mathrm{A7}}\left(1 - e^{-\mu_{\mathrm{B,A}}\left(t_{\mathrm{A1}}-\frac{V_{\mathrm{P}}}{F}\right)}\right) + M_{\mathrm{A8}}\left(t_{\mathrm{A1}}-\frac{V_{\mathrm{P}}}{F}\right) + M_{\mathrm{A9}}\left(t_{\mathrm{A1}}-\frac{V_{\mathrm{P}}}{F}\right)e^{-\mu_{\mathrm{B,A}}\left(t_{\mathrm{A1}}-\frac{V_{\mathrm{P}}}{F}\right)}\Big\}u\left(t_{\mathrm{A1}}-\frac{V_{\mathrm{P}}}{F}\right) + \Big\{M_{\mathrm{A10}} + M_{\mathrm{A11}}e^{-\mu_{\mathrm{B,A}}\left(t_{\mathrm{A2}}-\frac{V_{\mathrm{P}}}{F}\right)} + M_{\mathrm{A12}}e^{-\mu_{\mathrm{G,A}}\left(t_{\mathrm{A2}}-\frac{V_{\mathrm{P}}}{F}\right)} + M_{\mathrm{A13}}\left(t_{\mathrm{A2}}-\frac{V_{\mathrm{P}}}{F}\right) + M_{\mathrm{A14}}\left(t_{\mathrm{A2}}-\frac{V_{\mathrm{P}}}{F}\right)e^{-\mu_{\mathrm{B,A}}\left(t_{\mathrm{A2}}-\frac{V_{\mathrm{P}}}{F}\right)}\Big\}u\left(t_{\mathrm{A2}}-\frac{V_{\mathrm{P}}}{F}\right)\Bigg], \tag{14}$$

where $M_{\mathrm{A1}} = L_{\mathrm{A1}}$, $M_{\mathrm{A2}} = L_{\mathrm{A2}}$, $M_{\mathrm{A3}} = L_{\mathrm{A3}}$, $M_{\mathrm{A4}} = \frac{M_{\mathrm{A1}}a_{\mathrm{G,A}}}{\mu_{\mathrm{B,A}}-\mu_{\mathrm{G,A}}}\left(1 + \frac{\mu_{\mathrm{B,A}}}{\mu_{\mathrm{B,A}}-\mu_{\mathrm{G,A}}}\right)$, $M_{\mathrm{A5}} = -M_{\mathrm{A3}}\left(\frac{\mu_{\mathrm{B,A}}}{\mu_{\mathrm{B,A}}-\mu_{\mathrm{G,A}}}\right)^2$, $M_{\mathrm{A6}} = -\frac{M_{\mathrm{A2}}a_{\mathrm{G,A}}}{\mu_{\mathrm{B,A}}-\mu_{\mathrm{G,A}}}$, $M_{\mathrm{A7}} = -M_{\mathrm{A1}}M_1\left(1 - \frac{2M_2}{\mu_{\mathrm{B,A}}}\right)$, $M_{\mathrm{A8}} = -M_{\mathrm{A1}}M_1M_2$, $M_{\mathrm{A9}} = M_{\mathrm{A1}}M_1(\mu_{\mathrm{B,A}} - M_2)$, $M_{\mathrm{A10}} = -M_{\mathrm{A3}}M_1\left\{1 - M_2\left(\frac{2}{\mu_{\mathrm{B,A}}} + \frac{1}{\mu_{\mathrm{G,A}}}\right)\right\}$, $M_{\mathrm{A11}} = -\frac{M_{\mathrm{A6}}M_1}{\mu_{\mathrm{B,A}}{}^2}\left\{(\mu_{\mathrm{B,A}} - M_2)\left(1 + \frac{\mu_{\mathrm{B,A}}}{\mu_{\mathrm{B,A}}-\mu_{\mathrm{G,A}}}\right) - M_2\right\}$, $M_{\mathrm{A12}} = -\frac{M_{\mathrm{A5}}M_1}{\mu_{\mathrm{G,A}}}(\mu_{\mathrm{G,A}} - M_2)$, $M_{\mathrm{A13}} = -M_{\mathrm{A3}}M_1M_2$, and $M_{\mathrm{A14}} = -\frac{M_{\mathrm{A6}}M_1}{\mu_{\mathrm{B,A}}}(\mu_{\mathrm{B,A}} - M_2)$, where $M_1 = e^{-\frac{PS}{F}}$ and $M_2 = \frac{v_{\mathrm{P}}}{v_{\mathrm{I}}}\frac{PS}{V_{\mathrm{P}}}\frac{PS}{F}$,

$$C_{\mathrm{T,A,TK}}(t) = \frac{F}{V_{\mathrm{T}}}\frac{1}{1-H_{\mathrm{LV}}}[\{N_{\mathrm{A1}}(e^{-N_1t_{\mathrm{A1}}} - e^{-\mu_{\mathrm{B,A}}t_{\mathrm{A1}}}) + N_{\mathrm{A2}}t_{\mathrm{A1}}e^{-\mu_{\mathrm{B,A}}t_{\mathrm{A1}}}\}u(t_{\mathrm{A1}}) + \{N_{\mathrm{A3}}e^{-N_1t_{\mathrm{A2}}} + \tag{15}$$

$N_{A4}e^{-\mu_{B,A}t_{A2}} + N_{A5}e^{-\mu_{G,A}t_{A2}} + N_{A6}t_{A2}e^{-\mu_{B,A}t_{A2}}\}u(t_{A2})],$

where $N_{A1} = \frac{Ea_{B,A}}{(\mu_{B,A}-N_1)^2}$, $N_{A2} = -N_{A1}(\mu_{B,A} - N_1)$, $N_{A3} = \frac{N_{A1}a_{G,A}}{\mu_{G,A}-N_1}$, $N_{A4} = \frac{N_{A3}(\mu_{G,A}-N_1)}{\mu_{B,A}-\mu_{G,A}}\left(1 + \frac{\mu_{B,A}-N_1}{\mu_{B,A}-\mu_{G,A}}\right)$, $N_{A5} = -N_{A3}\left(\frac{\mu_{B,A}-N_1}{\mu_{B,A}-\mu_{G,A}}\right)^2$, and $N_{A6} = -\frac{N_{A5}(\mu_{G,A}-N_1)(\mu_{B,A}-\mu_{G,A})}{\mu_{B,A}-N_1}$, where $N_1 = \frac{v_P}{v_I}\frac{EF}{V_P}$,

$$C_{T,A,ETK}(t) = v_P \frac{C_A(t-t_{Lag,T})}{1-H_{LV}} + C_{T,A,TK}(t), \quad (16)$$

where $C_{T,A,TK}(t)$, $C_{T,A,ETK}(t)$, $C_{T,A,2CX}(t)$, $C_{T,A,AATH}(t)$ and $C_{T,A,DP}(t)$ are $C_{T,A}(t)$ for the TK, ETK, 2CX, AATH and DP models, respectively. It should be recognized that the analytic solution of $C_{T,PA}(t)$ is of the same form as that of $C_{T,A}(t)$ because the AIF model is of the same form between them.

D. Parameter calculation

The number and type of curve-fitting parameters were the same among the five different models: F/V_P, γ, PS/V_P, v_P, v_I, and $t_{Lag,T}$. The BF, BF_{PA}, BF_A, BV, MTT, PS, and K^{Trans} can be computed according to: $BV = 100 \cdot \frac{V_P}{(1-H_{SV})m} = 100 \cdot \frac{v_P}{(1-H_{SV})\rho_T}$ (in mL/100 g), where H_{SV} is the hematocrit in small vessels (≅0.25) and $m = \rho_T V_T$ is the mass of the tissue with density ρ_T (1.04 g/cm^3 in the case of soft tissue), $BF = BV \cdot \frac{F}{V_P}$ (in mL/min/100 g), $BF_{PA} = \gamma BF$ (in mL/min/100 g), $BF_A = (1-\gamma)BF$ (in mL/min/100 g), $MTT = \frac{V_P+V_I}{F}$ ($MTT = \frac{V_I}{F}$ for the TK model), where $V_I = v_I V_T$ is the interstitial volume (in mL), $PS = (1-H_{SV}) \cdot BV \cdot \frac{PS}{V_P}$ (in mL/min/100 g) and $K^{Trans} = E \cdot \frac{F}{V_T} = E \cdot \frac{F}{V_P} \cdot v_P$ (in mL/min/mL).

E. Multimodel comparison

The five different tracer kinetic models described above form a set of candidate models to quantify DCE data. The AIC and the associated Akaike weights, w_m, rank different models on the basis of goodness-of-fit and number of parameters. The AIC provides an objective relative measure for the information lost when approximating real data with a model. For each voxel, the cAIC for small sample sizes is given by

$$cAIC = -2\log\mathcal{L} + 2K\left(\frac{N}{N-K-1}\right), \tag{17}$$

where $\log\mathcal{L} = -\frac{N}{2}[\ln(2\pi\hat{\sigma}^2) + 1]$ is the maximized log likelihood with N the sample size (i.e., the number of temporal data points) and $\hat{\sigma}^2 = RSS/N$ the normalized residual sum of squares [4]. The number of the estimated model parameters (including $\hat{\sigma}^2$) is K. It is advocated to use the cAIC when the ratio N/K is small (<40). Based on the cAIC values, for each voxel, the optimal model was chosen by selecting the $cAIC_{min}$. To assess the relative likelihood of a model, the cAIC differences (Δ_m) were calculated between models as

$$\Delta_m = cAIC_m - cAIC_{\min}, \tag{18}$$

with $cAIC_m$ being the cAIC value of candidate model m. The model estimated to be the best has $\Delta_m = 0$. For each model out of the set of M alternative models, the AW, w_m, was calculated from Δ_m:

$$w_m = \frac{\exp(-\Delta_m/2)}{\sum_{r=1}^{M}\exp(-\Delta_r/2)}. \tag{19}$$

The value of w_m represents the probability that a certain model is the best among M models.

References

1. Johnson JA, Wilson TA. A model for capillary exchange. *The American journal of physiology.* 1966;210(6):1299-1303.
2. Koh TS. On the a priori identifiability of the two-compartment distributed parameter model from residual tracer data acquired by dynamic contrast-enhanced imaging. *IEEE transactions on bio-medical engineering.* 2008;55(1):340-344.
3. Orton MR, d'Arcy JA, Walker-Samuel S, et al. Computationally efficient vascular input function models for quantitative kinetic modelling using DCE-MRI. *Phys Med Biol.* 2008;53(5):1225-1239.
4. Brix G, Zwick S, Kiessling F, Griebel J. Pharmacokinetic analysis of tissue microcirculation using nested models: multimodel inference and parameter identifiability. *Med Phys.* 2009;36(7):2923-2933.
