# Supplementary material for: Dual‐input tracer kinetic modeling of dynamic contrast‐enhanced MRI in thoracic malignancies
Source: J Appl Clin Med Phys. 2019 Oct 11;20(11):169–88. doi: 10.1002/acm2.12740 (PMC6839367; doi:10.1002/acm2.12740)
Supplement: Supplementary file 2 — SupInfo S2. Supplementary document 2. [file ACM2-20-169-s002.pdf]

Supplementary document 2

Summary of terms and parameters used in the five dual-input models described in this paper

Term	Definition	Unit of Measure
$C_{\mathrm{PA}}(t)$	Pulmonary arterial blood concentration of CA	g/mL
$C_{\mathrm{A}}(t)$	Systemic arterial blood concentration of CA	g/mL
$C_{\mathrm{T}}(t)$	Concentration of CA in tissue	g/mL
$R_{\mathrm{T}}(t)$	Tissue residue function	None
$Q_{\mathrm{T,PA}}(t)$	Pulmonary impulse response function of the tissue	mL/min/mL
$Q_{\mathrm{T,A}}(t)$	Systemic impulse response function of the tissue	mL/min/mL
$Q_{\mathrm{T}}(t)$	Total impulse response function of the tissue	mL/min/mL
F	Total pulmonary plasma flow	mL/min
γ	Pulmonary arterial flow fraction	None
F_{PA}	Pulmonary arterial plasma flow	mL/min
F_{A}	Systemic plasma flow	mL/min
BF	Total pulmonary blood flow	mL/min/100 g
BF_{PA}	Pulmonary arterial blood flow	mL/min/100 g
BF_{A}	Systemic arterial blood flow	mL/min/100 g
BV	Blood volume	mL/100 g
MTT	Mean transit time	min
PS	Permeability-surface area product	mL/min (or mL/min/100 g)
v_{P}	Fractional plasma volume	None
v_{I}	Fractional interstitial volume	None
E	Extraction fraction	None
H_{LV}	Hematocrit in major (large) vessels	None
H_{SV}	Hematocrit in small vessels	None
m	Tissue mass	g
ρ_{T}	Tissue density	g/cm^3
V_{P}	Volume of the plasma compartment	mL
V_{I}	Volume of the interstitial compartment	mL
V_{T}	Tissue volume	mL
F/V_{T}	Total pulmonary perfusion	mL/min/mL
$F_{\mathrm{PA}}/V_{\mathrm{T}}$	Pulmonary arterial perfusion	mL/min/mL
$F_{\mathrm{A}}/V_{\mathrm{T}}$	Systemic arterial perfusion	mL/min/mL
$K^{\mathrm{Trans}} = EF/V_{\mathrm{T}}$	Volume transfer constant between the plasma and interstitial compartments	mL/min/mL

V_P/F	Capillary transit time	min
PS/F	Capillary leakage time	min
$t_{Lag,T}$	Difference in bolus arrival time between $C_{PA}(t)$ (or $C_A(t)$) and $C_T(t)$	min
